# Employment outcomes of people with Long Covid symptoms: community-based cohort study

**DOI:** 10.1093/eurpub/ckae034

**Published:** 2024-02-29

**Authors:** Daniel Ayoubkhani, Francesco Zaccardi, Koen B Pouwels, A Sarah Walker, Donald Houston, Nisreen A Alwan, Josh Martin, Kamlesh Khunti, Vahé Nafilyan

**Affiliations:** Leicester Real World Evidence Unit, Leicester Diabetes Centre, Department of Population Health Sciences, University of Leicester, Leicester, UK; Data and Analysis for Social Care and Health Division, Office for National Statistics, Newport, UK; Leicester Real World Evidence Unit, Leicester Diabetes Centre, Department of Population Health Sciences, University of Leicester, Leicester, UK; National Institute for Health Research Health Protection Research Unit in Healthcare Associated Infections and Antimicrobial Resistance, University of Oxford, Oxford, UK; Health Economics Research Centre, Nuffield Department of Population Health, University of Oxford, Oxford, UK; National Institute for Health Research Health Protection Research Unit in Healthcare Associated Infections and Antimicrobial Resistance, University of Oxford, Oxford, UK; Nuffield Department of Medicine, University of Oxford, Oxford, UK; City-Regional Economic Development Institute, Birmingham Business School, University of Birmingham, Birmingham, UK; School of Primary Care, Population Sciences and Medical Education, Faculty of Medicine, University of Southampton, Southampton, UK; NIHR Southampton Biomedical Research Centre, University of Southampton and University Hospital Southampton NHS Foundation Trust, Southampton, UK; NIHR Applied Research Collaboration (ARC) Wessex, Southampton, UK; Bank of England, London, UK; Leicester Real World Evidence Unit, Leicester Diabetes Centre, Department of Population Health Sciences, University of Leicester, Leicester, UK; Data and Analysis for Social Care and Health Division, Office for National Statistics, Newport, UK; Department of Medical Statistics, Faculty of Epidemiology and Population Health, Environment and Society, London School of Hygiene & Tropical Medicine, London, UK

## Abstract

**Background:**

Evidence on the long-term employment consequences of SARS-CoV-2 infection is lacking. We used data from a large, community-based sample in the UK to estimate associations between Long Covid and employment outcomes.

**Methods:**

This was an observational, longitudinal study using a pre–post design. We included survey participants from 3 February 2021 to 30 September 2022 when they were aged 16–64 years and not in education. Using conditional logit modelling, we explored the time-varying relationship between Long Covid status ≥12 weeks after a first test-confirmed SARS-CoV-2 infection (reference: pre-infection) and labour market inactivity (neither working nor looking for work) or workplace absence lasting ≥4 weeks.

**Results:**

Of 206 299 participants (mean age 45 years, 54% female, 92% white), 15% were ever labour market inactive and 10% were ever long-term absent during follow-up. Compared with pre-infection, inactivity was higher in participants reporting Long Covid 30 to <40 weeks [adjusted odds ratio (aOR): 1.45; 95% CI: 1.17–1.81] or 40 to <52 weeks (aOR: 1.34; 95% CI: 1.05–1.72) post-infection. Combining with official statistics on Long Covid prevalence, and assuming a correct statistical model, our estimates translate to 27 000 (95% CI: 6000–47 000) working-age adults in the UK being inactive because of Long Covid in July 2022.

**Conclusions:**

Long Covid is likely to have contributed to reduced participation in the UK labour market, though it is unlikely to be the sole driver. Further research is required to quantify the contribution of other factors, such as indirect health effects of the pandemic.

## Introduction

A proportion of people infected with severe acute respiratory syndrome coronavirus 2 (SARS-CoV-2) continue to experience symptoms months or years later, known as Long Covid, post coronavirus disease 2019 (COVID-19) syndrome or post COVID-19 condition.

In the UK, post COVID-19 syndrome is clinically defined as ‘signs and symptoms that develop during or after an infection consistent with COVID-19, continue for more than 12 weeks and are not explained by an alternative diagnosis’.^1^ Internationally, the World Health Organisation defines post COVID-19 condition as ‘occurring in individuals with a history of probable or confirmed SARS-CoV-2 infection, usually 3 months from the onset of COVID-19 with symptoms and that last for at least 2 months and cannot be explained by an alternative diagnosis’.^2^

Common Long Covid symptoms include fatigue, breathlessness, muscle/joint pain, and cognitive impairment.^3–5^ A substantial proportion of people with Long Covid also report psychiatric symptoms associated with depression and anxiety,^6^ although these may not be a direct result of past SARS-CoV-2 infection.^7^^,^^8^ In March 2023, an estimated 1.9 million people in the UK (2.9% of the population) self-reported Long Covid, with prevalence being highest among working-age people.^6^

The growing number of SARS-CoV-2 infections in the UK has coincided with rising labour market inactivity, defined as neither being in work nor actively seeking it. By the end of 2022, the number of working-age people who were inactive had increased by over 350 000 since pre-pandemic, with inactivity due to long-term sickness growing most quickly.^9^ One study suggests that nearly half of UK firms had employees with Long Covid in 2022, and a quarter reported it as a substantial cause of long-term absence.^10^ Long Covid may have resulted in long-term absence for 110 000 UK workers and the loss of 4.4 million working hours per week.^11^ This may have implications for the livelihood of individuals but also for the health of the macroeconomy, including income and earnings, labour market supply, productivity, tax receipts, benefit payments and consumer demand.

However, despite the potential impact of Long Covid on global labour markets, there is limited evidence on its relationship with employment. In one study, 45% of participants with Long Covid required a reduced work schedule and 22% were not working due to their illness seven months post-infection.^12^ In another analysis, 19% of participants with Long Covid were unable to work, 10% reported reduced working hours, and 37% said that their income had been affected.^13^ Among participants with SARS-CoV-2, Long Covid has been associated with 44% higher odds of not working and 27% lower odds of working full-time.^14^ A study of 50 000 participants with SARS-CoV-2 found an average reduction in self-reported working capacity of 10.7% after a mean follow-up of 8.5 months post-infection.^15^

While providing valuable insights, these studies are largely cross-sectional and descriptive in nature, lack robust control groups for comparison, and are based on convenience samples that may not be representative. Inferential studies on Long Covid and employment outcomes are scarce, and limited sample sizes and follow-up time have precluded detailed analysis of population subgroups. Therefore, in this study, we used longitudinal data from a large, community-based sample to estimate associations between Long Covid and employment outcomes.

## Methods

### Study design and data

We performed an observational, longitudinal, pre–post analysis of participants from the UK COVID-19 Infection Survey (CIS, ISRCTN21086382),^16^ a study of people aged ≥2 years from randomly sampled households (excluding hospitals, care homes, halls of residence and prisons). The CIS received ethical approval from the South Central Berkshire B Research Ethics Committee (20/SC/0195). Enrolment took place from 26 April 2020 to 31 January 2022, accruing over 530 000 participants from over 260 000 households. Supplementary table S1 reports household enrolment rates, which were as high as 51% at the start of the survey (comprising previous respondents to government surveys who had consented to participate in future research) but dropped to 12% by the end of recruitment (when sampling was conducted randomly from national address lists; the majority of participating households were recruited during this phase); >97% of enrolled participants consented to monthly assessments for ≥1 year.

Participants provided a self-swab for polymerase chain reaction (PCR) testing at each assessment, while a sub-sample also provided blood samples for antibody testing. Participants reported whether they had tested positive for SARS-CoV-2 or antibodies outside of the study and their current Long Covid status (‘Would you describe yourself as having ‘Long Covid’, that is, you are still experiencing symptoms more than 4 weeks after you first had COVID-19 that are not explained by something else?’). Data collection was conducted via face-to-face interviews until June 2022 before switching to remote (online or telephone) collection.

### Inclusion and exclusion criteria

We included monthly assessments from 3 February 2021 (when Long Covid data were first collected) to 30 September 2022 when participants responded to the Long Covid question, were aged 16–64 years, and were not in full-time education.

We excluded participants with a first positive swab for SARS-CoV-2 (either a PCR test via study assessments or any self-reported test outside of the study) at enrolment, as the timing of infection could not be determined for these participants. We also excluded participants with a positive spike-antibody test (excluding any tests after COVID-19 vaccination) or who suspected they had COVID-19 ≥ 14 days before their first positive swab, as the latter may represent a reinfection. To ensure we could fully observe participants’ self-reported Long Covid experience, we excluded participants first testing positive before 11 November 2020 (12 weeks before Long Covid data started being collected).

When analysing long-term absence, we excluded assessments when participants were not in employment, as well as those before 1 October 2021 (when the UK Coronavirus Job Retention Scheme was in operation).

### Exposures

The time-varying exposure was past SARS-CoV-2 infection (determined from CIS and self-reported swabs) by current Long Covid status (positive responses to the Long Covid question ≥12 weeks after a first positive swab): uninfected, infected <12 weeks ago, infected ≥12 weeks ago without reporting Long Covid to date, infected ≥12 weeks ago and currently reporting Long Covid, and infected ≥12 weeks ago and previously reported Long Covid. Participants infected ≥12 weeks ago were stratified by time since first positive test.

### Outcomes

The outcomes were labour market inactivity (excluding retirement, i.e. neither working nor looking for work, and not retired); and workplace absence for ≥4 weeks for any reason whilst in employment. The CIS question used to derive these outcomes can be found in Supplementary Appendix S1.

### Covariates

We considered a range of socio-demographic variables hypothesized to be related to both Long Covid and employment status: age, sex, white or non-white ethnicity, country/region, area deprivation quintile group, and self-reported health/disability status. We also examined labour market variables: employment status, employment sector, occupation group and whether self-employed. All variables were measured at CIS enrolment and thus before any SARS-CoV-2 infection.

### Statistical methods

We descriptively compared confounders between participants who ever or never reported Long Covid during follow-up using means and proportions for continuous and categorical variables, respectively. Large differences between groups were indicated by absolute standardized differences >10%, a threshold widely used in previous research.^17^

We fitted conditional logit (fixed effects) models controlling for background labour market conditions over the study period by adjusting for the calendar day of each study assessment (modelled as a restricted cubic spline with boundary knots at the 10th and 90th percentiles and an internal knot at the median of the time distribution) interacted with current age (restricted cubic spline, as for calendar day), sex and health/disability status at CIS enrolment. We performed several sensitivity analyses for the primary outcome (labour market inactivity), detailed in Supplementary Appendix S2. A range of heterogeneity tests for both study outcomes can be found in Supplementary Appendix S3.

Models were fitted using the ‘clogit’ function in R’s ‘survival’ package.^18^ We reported results as adjusted odds ratios (aORs) and 95% confidence intervals (CIs), with pre-infection being the reference group. All statistical analyses were performed using R version 4.0.

### Population attributable risk

By combining our aORs with official statistics on the population prevalence of Long Covid by inactivity status and time since infection,^19^ we estimated the number of working-age adults in the UK who were inactive in July 2022 because of Long Covid (assuming a correct statistical model); that is, those reporting Long Covid who would have been working had they not been infected with SARS-CoV-2. A detailed description of these calculations can be found in Supplementary Appendix S4.

## Results

### Characteristics of study participants

The analysed population comprised 206 299 participants (Supplementary figure S1) contributing a total of 2 547 618 monthly follow-up assessments (mean 12.3 assessments per participant). 147 895 participants (contributing 1 155 207 assessments) were in employment from 1 October 2021 and were therefore included in the analysis of long-term absence. 97 751 participants (47.4% of the total analysed population of 206 299) tested positive for SARS-CoV-2 during follow-up, while the remaining 108 548 (52.6% of the total) never tested positive. Of the 97 751 participants who tested positive, 8440 (8.6%) reported Long Covid ≥12 weeks post-infection.

Compared with participants infected with SARS-CoV-2 without reporting Long Covid, those who reported Long Covid were on average older (46.3 vs. 44.3 years) at enrolment, and were more likely to be female (63.2% vs. 55.1%), living in the most deprived quintile group (14.9% vs. 10.8%), living with a long-term health condition/disability (24.2% vs. 16.2%), and inactive in the labour market (9.7% vs. 6.6%) (table 1). Among infected participants who were employed at enrolment, those who reported Long Covid during follow-up were more likely to be working in the teaching and education sector (16.0% vs. 12.8%) and in caring, leisure and other service occupations (10.3% vs. 6.5%). A comparison of study participants who reported Long Covid versus those not infected with SARS-CoV-2 can be found in Supplementary table S2.

**Table 1:** Characteristics at enrolment of study participants ever infected with SARS-CoV-2 during follow-up, stratified by whether participants ever subsequently reported Long Covid

Characteristic	Level	**All infected participants (*n*** **=** **97** **751)**	**Never reported Long Covid (*n*** **=** **89** **311)**	**Ever reported Long Covid (*n*** **=** **8440)**	Absolute standardized difference (%)
Age (years), mean (SD)		44.5 (12.3)	44.3 (12.3)	46.3 (11.2)	16.6
Age group (*n*, %)	<16 years	489 (0.5)	474 (0.5)	15 (0.2)	20.8
	16–24 years	6574 (6.7)	6197 (6.9)	377 (4.5)	
	25–34 years	15 151 (15.5)	14 176 (15.9)	975 (11.6)	
	35–49 years	36 284 (37.1)	32 984 (36.9)	3300 (39.1)	
	50–64 years	39 253 (40.2)	35 480 (39.7)	3773 (44.7)	
Sex (*n*, %)	Male	43 201 (44.2)	40 091 (44.9)	3110 (36.8)	16.4
	Female	54 550 (55.8)	49 220 (55.1)	5330 (63.2)	
Ethnic group (*n*, %)	White	90 200 (92.3)	82 326 (92.2)	7874 (93.3)	4.3
	Non-white	7551 (7.7)	6985 (7.8)	566 (6.7)	
Country/region of residence (*n*, %)	North East England	3549 (3.6)	3160 (3.5)	389 (4.6)	13.6
	North West England	11 135 (11.4)	10 073 (11.3)	1062 (12.6)	
	Yorkshire and the Humber	8088 (8.3)	7349 (8.2)	739 (8.8)	
	East Midlands	6142 (6.3)	5546 (6.2)	596 (7.1)	
	West Midlands	7061 (7.2)	6388 (7.2)	673 (8.0)	
	East of England	8723 (8.9)	7921 (8.9)	802 (9.5)	
	London	17 253 (17.6)	16 029 (17.9)	1224 (14.5)	
	South East England	11 853 (12.1)	10 922 (12.2)	931 (11.0)	
	South West England	7482 (7.7)	6876 (7.7)	606 (7.2)	
	Scotland	8268 (8.5)	7590 (8.5)	678 (8.0)	
	Wales	4897 (5.0)	4442 (5.0)	455 (5.4)	
	Northern Ireland	3300 (3.4)	3015 (3.4)	285 (3.4)	
Area deprivation quintile group (*n*, %)	1 (most deprived)	10 889 (11.1)	9629 (10.8)	1260 (14.9)	14.7
	2	16 510 (16.9)	14 889 (16.7)	1621 (19.2)	
	3	20 408 (20.9)	18 645 (20.9)	1763 (20.9)	
	4	23 441 (24.0)	21 608 (24.2)	1833 (21.7)	
	5 (least deprived)	26 503 (27.1)	24 540 (27.5)	1963 (23.3)	
Self-reported health/disability status (*n*, %)	No long-term health conditions	81 211 (83.1)	74 813 (83.8)	6398 (75.8)	20.4
	Health conditions without impact to day-to-day activities	9358 (9.6)	8422 (9.4)	936 (11.1)	
	Day-to-day activities limited a little by health conditions	4572 (4.7)	3906 (4.4)	666 (7.9)	
	Day-to-day activities limited a lot by health conditions	2610 (2.7)	2170 (2.4)	440 (5.2)	
Employment status (*n*, %)	Employed	79 121 (80.9)	72 356 (81.0)	6765 (80.2)	16.8
	Unemployed	1885 (1.9)	1706 (1.9)	179 (2.1)	
	Not working and not looking for work	6759 (6.9)	5939 (6.6)	820 (9.7)	
	Retired	6747 (6.9)	6209 (7.0)	538 (6.4)	
	Student	3239 (3.3)	3101 (3.5)	138 (1.6)	
Employment sector, among participants in employment (*n*, %)	Teaching and education	10 374 (13.1)	9289 (12.8)	1085 (16.0)	14.1
	Health care	7315 (9.2)	6676 (9.2)	639 (9.4)	
	Social care	1988 (2.5)	1782 (2.5)	206 (3.0)	
	Transport	2527 (3.2)	2305 (3.2)	222 (3.3)	
	Retail and wholesale	4781 (6.0)	4370 (6.0)	411 (6.1)	
	Hospitality	2040 (2.6)	1846 (2.6)	194 (2.9)	
	Food production, agriculture and farming	1150 (1.5)	1053 (1.5)	97 (1.4)	
	Personal services	856 (1.1)	786 (1.1)	70 (1.0)	
	Information technology and communication	4906 (6.2)	4604 (6.4)	302 (4.5)	
	Financial services	5795 (7.3)	5430 (7.5)	365 (5.4)	
	Manufacturing and construction	6793 (8.6)	6228 (8.6)	565 (8.4)	
	Civil service and local government	5031 (6.4)	4540 (6.3)	491 (7.3)	
	Armed forces	250 (0.3)	232 (0.3)	18 (0.3)	
	Arts, entertainment and recreation	1599 (2.0)	1479 (2.0)	120 (1.8)	
	Other	10 305 (13.0)	9487 (13.1)	818 (12.1)	
	Unknown	13 411 (16.9)	12 249 (16.9)	1162 (17.2)	
SOC Major Group, among participants in employment (*n*, %)	Managers, directors and senior officials	6990 (8.8)	6456 (8.9)	534 (7.9)	18.4
	Professional occupations	18 521 (23.4)	17 143 (23.7)	1378 (20.4)	
	Associate professional and technical occupations	12 488 (15.8)	11 509 (15.9)	979 (14.5)	
	Administrative and secretarial occupations	8903 (11.3)	8093 (11.2)	810 (12.0)	
	Skilled trades occupations	4894 (6.2)	4457 (6.2)	437 (6.5)	
	Caring, leisure and other service occupations	5421 (6.9)	4727 (6.5)	694 (10.3)	
	Sales and customer service occupations	3380 (4.3)	3065 (4.2)	315 (4.7)	
	Process, plant and machine operatives	2380 (3.0)	2166 (3.0)	214 (3.2)	
	Elementary occupations	3253 (4.1)	2927 (4.0)	326 (4.8)	
	Unknown	12 891 (16.3)	11 813 (16.3)	1078 (15.9)	
Self-employment status, among participants in employment (*n*, %)	Employee	72 616 (91.8)	66 412 (91.8)	6204 (91.7)	0.3
	Self-employed	6505 (8.2)	5944 (8.2)	561 (8.3)	

Notes: SD, standard deviation; SOC, standard occupational classification. Area deprivation was based on the English Indices of Deprivation 2019, the Welsh Index of Multiple Deprivation 2019, the Scottish Index of Multiple Deprivation 2020 and the Northern Ireland Multiple Deprivation Measure 2017.

### Labour market inactivity

A total of 31 248 study participants (15.1%) were ever inactive during follow-up. Irrespective of timing, 17.7% of participants who ever reported Long Covid were ever inactive, compared with 13.4% of those infected with SARS-CoV-2 without reporting Long Covid.

**Figure 1:**
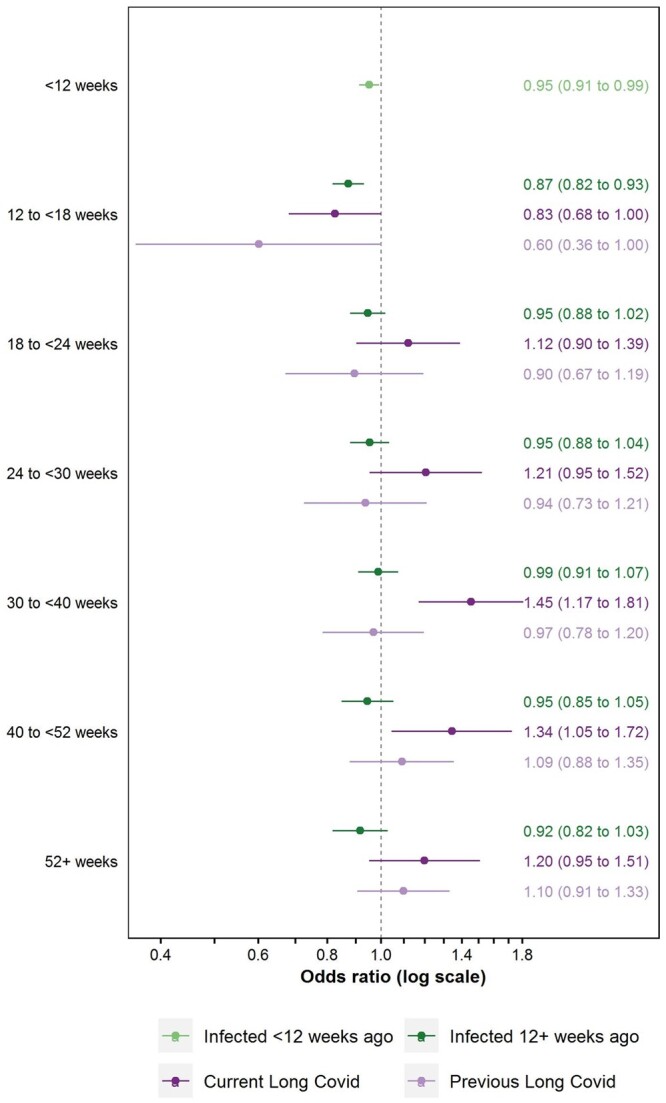
Adjusted odds ratios for inactivity (excluding retirement) compared with the pre-infection period, by time since SARS-CoV-2 infection and Long Covid status. Notes: Estimates are from a conditional logit model adjusted for calendar day of study assessment, current age, and interactions between calendar day and each of current age, sex, and self-reported health/disability status at survey enrolment

Compared with pre-infection, inactivity was less common in the first 12 weeks [aOR: 0.95 (95% CI: 0.91–0.99)] and 12 to <18 weeks post-infection [aOR: 0.87 (95% CI: 0.82–0.93)] without reporting Long Covid; aOR: 0.83 (95% CI: 0.68–1.00) while reporting Long Covid; aOR: 0.60 (95% CI: 0.36–1.00) previously reported Long Covid) (figure 1). Beyond 18 weeks post-infection, there was no evidence of differences in the odds of inactivity compared with pre-infection for participants who had not reported Long Covid to date or had previously reported Long Covid. Conversely, participants currently reporting Long Covid 30 to <40 or 40 to <52 weeks post-infection were more likely to be inactive compared with pre-infection, with aORs of 1.45 (95% CI: 1.17–1.81) and 1.34 (95% CI: 1.05–1.72), respectively.

Applying these aORs to published official statistics on the population prevalence of Long Covid by inactivity status and time since infection, an estimated 27 000 (95% CI: 6000–47 000) working-age non-students were inactive because of Long Covid in July 2022, assuming a correct statistical model.

In sensitivity analysis, similar results were obtained when restricting the population to participants testing positive for SARS-CoV-2, excluding assessments when participants were retired, and increasing the number of internal knots in the splines for calendar time and age (Supplementary Appendix S2). Supplementary figure S2 demonstrates the benefit of adjusting for calendar time compared with unadjusted estimates.

### Long-term absence

Of 147 895 participants in employment from 1 October 2021, 14 493 (9.8%) ever reported long-term absence. Irrespective of timing, long-term absence was experienced by 13.1% of participants who reported Long Covid, compared with 9.8% of those infected with SARS-CoV-2 without reporting Long Covid.

**Figure 2:**
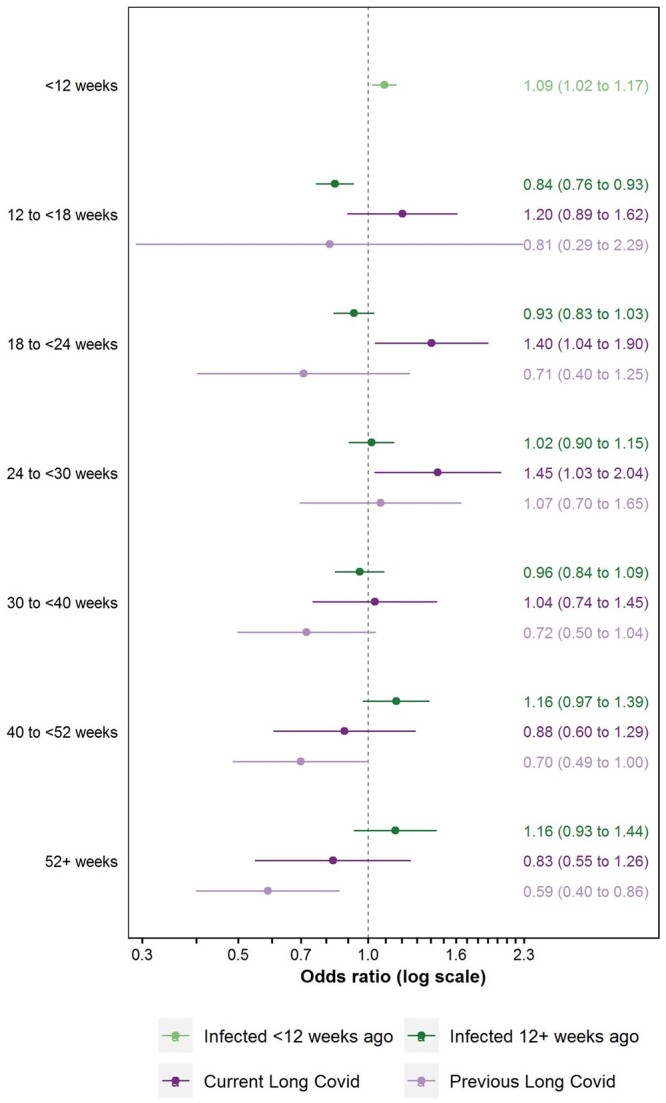
Adjusted odds ratios for long-term (≥4 weeks) absence compared with the pre-infection period, by time since SARS-CoV-2 infection and Long Covid status. Notes: Estimates are from a conditional logit model adjusted for calendar day of study assessment, current age, and interactions between calendar day and each of current age, sex, and self-reported health/disability status at survey enrolment. The model was fitted to study assessments from 1 October 2021 when participants were in employment

Compared with pre-infection, SARS-CoV-2 infection <12 weeks previously was associated with an increased likelihood of long-term absence [aOR: 1.09 (95% CI: 1.02–1.17)]; as too was reporting Long Covid 18 to <24 or 24 to <30 weeks post-infection, with aORs of 1.40 (95% CI: 1.04–1.90) and 1.45 (95% CI: 1.03–2.04), respectively (figure 2). Conversely, infection 12 to <18 weeks previously without reporting Long Covid to date [aOR: 0.84 (95% CI: 0.76–0.93)], or being 40 to <52 weeks [aOR: 0.70 (95% CI: 0.49–1.00)] or ≥52 weeks [aOR: 0.59 (95% CI: 0.40–0.86)] post-infection having previously reported Long Covid, were both associated with reduced odds of long-term absence relative to pre-infection.

## Discussion

### Findings in context

The main finding of our study is that reporting Long Covid after SARS-CoV-2 infection is associated with increased odds of labour market inactivity and long-term absence compared with pre-infection, with the period of greatest excess risk being 30 to <40 weeks post-infection for inactivity and 18 to <30 weeks post-infection for absence. Our results add to those from a limited number of longitudinal studies examining the employment outcomes of COVID-19, all with smaller sample sizes and shorter follow-up, from which the findings to date are mixed. Among 17 000 UK participants recruited via social and traditional media, SARS-CoV-2 was associated with a five-fold increase in the odds of sickness absence beyond the acute phase of infection, but there was no evidence of a relationship between COVID-19 and inadequate household income in the long-term.^20^ Among 36 000 UK Household Longitudinal Study (UKHLS) respondents, Long Covid was associated with reduced working hours but not being out of employment, with the former appearing to dissipate after six months.^11^

Our findings are also coherent with a small body of research internationally. In the US, Long Covid has been shown to be associated with a greater likelihood of unemployment and a reduced likelihood of working full-time.^14^ Participants in a German study experienced reduced working capacity 6–12 months after SARS-CoV-2 infection.^15^ Mean work ability scores were lower in a Swiss cohort with Long Covid 1-year post-infection compared with those without Long Covid.^21^

Our findings are coherent with the broader labour market landscape in the UK, which has been characterized by rising levels of inactivity throughout the pandemic.^22^ However, it seems unlikely that Long Covid is the sole, or even main, driver of this trend. The number of working-age adults who were inactive due to ill-health had been gradually rising since early 2019, nearly a year before the emergence of COVID-19.^23^ Furthermore, persistently increasing levels of inactivity during the pandemic has not been commonplace internationally, despite Long Covid having a global burden.^4^ The UK is among only 9 of 38 Organisation for Economic Co-operation and Development (OECD) member states for which the inactivity rate among people aged 15–64 years was higher in the third quarter of 2022 than three years earlier, and one of only six for which the rate continued to rise over the latest four quarters.^24^

To contextualize our estimated attributable risk of 27 000 people inactive because of Long Covid, this represents 0.5% of total inactivity (excluding retirement) among working-age non-students in the UK in July 2022,^22^ and 13% of those reporting Long Covid.^19^ This suggests the majority of inactive people with Long Covid may have been absent from the labour market even if they had not been infected with SARS-CoV-2, for example due to comorbidities. Other studies suggest that 80 000^25^–96 000^26^ people might have left employment directly because of Long Covid by March 2022. These numbers are higher than our estimate as they represent cumulative exits from employment rather than point-in-time measures.

We observed an inverted U-shaped relationship between Long Covid and inactivity over time, with the odds ratios peaking at 30 to <40 weeks post-infection before subsiding thereafter. This observation, coupled with lack of evidence of a relationship between time since infection and inactivity for individuals who previously had Long Covid symptoms, suggests that some people who left employment while experiencing Long Covid may have later returned to work (either in remission or with residual symptoms). This may have been facilitated by there being 1.2 million vacancies in the UK during the fourth quarter of 2022, 40% more than pre-pandemic.^27^ The rate of consumer price inflation was also high in the UK during 2022, peaking at 11.1% in October 2022 compared with 12 months earlier,^28^ thus financial pressures may have encouraged some people with Long Covid to return to work.

Our finding that the risk of absence levels off after 30 weeks post-infection may partly reflect people returning to work (including those with persistent illness) upon completion of their 28-week period of statutory sick pay. Continuing to work while sick, so called ‘presenteeism’, has been linked to reduced productivity^29^ and increased risk of future absence.^30^ However, we cannot rule out a survivorship effect: study participants remaining at risk of long-term absence beyond 30 weeks post-infection were those who had not yet left the workforce, including due to ill-health.

### Strengths and limitations

To the authors’ knowledge, this is the largest longitudinal study to examine the relationship between Long Covid and employment outcomes, with over 200 000 participants included in the analysis. Selection bias was minimized by selecting households at random from national address lists, while prospective data collection meant that responses were not affected by recall effects. The CIS enrolment rate had dropped to 12% by the end of recruitment, so possible selection effects mean that our findings are not necessarily generalizable to the broader population. However, the attrition rate was low, so our analysis is unlikely to have been substantially affected by loss-to-follow-up bias.

The conditional logit modelling approach implicitly controlled for all observed and unobserved time-invariant confounders, whereby each participant acted as their own control. However, we cannot rule out residual confounding by unmeasured time-varying factors (which may be evident from the observed negative association between SARS-CoV-2 infection and labour market inactivity <18 weeks post-infection), and reverse causality may have affected the estimated relationships between Long Covid and employment status to some extent. This means our estimate of labour market inactivity attributable to Long Covid, which relies on the assumption that the underlying statistical model is correct, should be interpreted with caution.

We did not have data on working hours, so we were unable to investigate the relationship between Long Covid and reduced working time. It was also not possible to distinguish long-term absence due to sickness from that due to other reasons, for example maternity/paternity leave. However, the latter are unlikely to be influenced by SARS-CoV-2, so we expect the impact on our findings to be small. Our estimates of inactivity due to Long Covid do not consider indirect impacts, such as family members of people with Long Covid reducing their working hours or leaving employment to take on caring responsibilities. Furthermore, our estimates do not include the effects of early retirement, transitioning to unemployment (not working but looking for work and available to start), or students choosing to remain in education instead of finding work. However, we expect these effects to be small because: there were 27 000 fewer working-age retirees in the UK in the final quarter of 2022 compared with 2019, before the pandemic;^22^ the unemployment rate in the UK dropped to 3.5% during 2022, a historic low since comparable records began in 1992;^22^ and the population prevalence of Long Covid is considerably lower among people aged <25 years (a group comprising the majority of students) than in older people.^6^ Future studies with longer follow-up could provide insights on a broader range of socio-economic outcomes following SARS-CoV-2 infection, such as income and earnings, social security benefit claims, and socio-economic position.

We excluded participants first testing positive for SARS-CoV-2 before 11 November 2020 and those with confirmed SARS-CoV-2 > 14 days before their first positive swab. This means that our findings are not necessarily generalizable to people who were infected in the ‘first wave’ of the COVID-19 pandemic (irrespective of whether they were later re-infected).

Long Covid status was self-reported by study participants, so exposure misclassification is possible (e.g. symptoms being caused by a medical condition unrelated to COVID-19). However, there is currently no biological test for Long Covid, so case ascertainment based on recorded clinical diagnoses is likely to lack sensitivity due to under-presentation, under-diagnosing and under-coding.^31^

## Conclusion

Long Covid is associated with labour market inactivity among working-age people, with the greatest risk occurring 30 to <40 weeks after SARS-CoV-2 infection. Long Covid is also associated with long-term absence 18 to <30 weeks post-infection. This reduced working capacity, and its downstream effects on income and living conditions, may be detrimental to the physical and mental health and wellbeing of the individuals affected.^32^ At a macroeconomic level, it is likely that Long Covid has contributed to reduced participation in the UK labour market to some extent, but it is unlikely to have been the sole driver, with fewer than 30 000 working-age adults estimated to be inactive because of Long Covid in July 2022. The contribution of factors besides Long Covid to reduced labour market participation, such as indirect health effects of the pandemic and extended healthcare waiting lists, remains unknown, and further research is required.

## Supplementary Material

ckae034_Supplementary_Data

## Data Availability

De-identified study data are available to accredited researchers in the ONS Secure Research Service (SRS) under part 5, chapter 5 of the Digital Economy Act 2017. For further information about accreditation, contact research.support@ons.gov.uk or visit: https://www.ons.gov.uk/aboutus/whatwedo/statistics/requestingstatistics/approvedresearcherscheme.

## Patient and public involvement

N.A.A. has lived experience of Long Covid.

Key points

- There is limited evidence on the relationship between Long Covid—symptoms persisting for months or years after a SARS-CoV-2 infection, estimated to affect nearly 3% of the UK population at the start of 2023—and subsequent changes in labour market participation.
- We used longitudinal data from a random sample of 206 299 working-age participants in the UK to estimate associations between Long Covid and labour market inactivity and long-term workplace absence from 3 February 2021 to 30 September 2022.
- Compared with pre-infection: the odds of labour market inactivity were 45% and 34% higher in participants reporting Long Covid 30 to <40 weeks or 40 to <52 weeks post-infection, respectively; and the odds of long-term workplace absence were 40% and 45% higher in participants reporting Long Covid 18 to <24 weeks or 24 to <30 weeks post-infection, respectively.
- Our estimates translate to 27 000 working-age adults in the UK being inactive because of their Long Covid symptoms in July 2022, representing approximately 0.5% of total labour market inactivity among the non-student, non-retired population.
- Long Covid is therefore likely to have contributed to reduced participation in the UK labour market, though it is unlikely to be the sole driver; further research is required to quantify the contribution of other factors, such as indirect health effects of the pandemic and extended healthcare waiting lists.
