# Supplementary material for: Employment outcomes of people with Long Covid symptoms: community-based cohort study
Source: Eur J Public Health. 2024 Feb 29;34(3):489–96. doi: 10.1093/eurpub/ckae034 (PMC11161149; doi:10.1093/eurpub/ckae034)
Supplement: ckae034_Supplementary_Data [file ckae034_supplementary_data.docx]

**Supplementary Table 1.** Number and percentage of households invited to participate in the COVID-19 Infection Survey who subsequently enrolled, by country and phase of study

| **Study phase** | **England** | **Wales** | **Northern Ireland** | **Scotland** |
| --- | --- | --- | --- | --- |
| Initial invitation | 10,266 (51%) | 7,031 (41%) | 7,373 (43%) | N/A |
| Extension period | 39,392 (43%) | N/A | N/A | N/A |
| AddressBase | 173,583 (12%) | 7,051 (14%) | N/A | 23,217 (13%) |

Notes: The initial invitation phase was open to previous respondents to ONS surveys who had consented to participate in future research, and started on 26 April 2020 in England, 29 June 2020 in Wales, and 26 July 2020 in Northern Ireland. The extension period refers to the period of time beyond the initial pilot phase of the study when the sample was increased, and started on 31 May 2020 in England. Sampling from AddressBase started on 13 July 2020 in England, 5 October 2020 in Wales, and 14 September 2020 in Scotland, and involved randomly selecting addresses from an address list. Enrolment rates are as of 31 January 2022, when recruitment into the study ended, and are taken from the technical dataset accompanying the official COVID-19 Infection Survey publication: https://www.ons.gov.uk/peoplepopulationandcommunity/healthandsocialcare/conditionsanddiseases/datasets/covid19infectionsurveytechnicaldata

**Supplementary Table 2.** Characteristics at enrolment of study participants who were either never infected with SARS-CoV-2 during follow-up, or who were infected and subsequently ever reported Long Covid

| **Characteristic** | **Level** | **All participants (*n*=****116,988)** | **Never infected (*n*=****108,548)** | **Ever reported Long Covid (*n*=****8,440)** | **Absolute standardized difference (%)** |
| --- | --- | --- | --- | --- | --- |
| Age (years), mean (SD) | | 45.2 (13.7) | 45.1 (13.9) | 46.3 (11.2) | 8.9 |
| Age group (*n*, %) | <16 years | 649 (0.6) | 634 (0.6) | 15 (0.2) | 37.9 |
|  | 16 to 24 years | 10,484 (9.0) | 10,107 (9.3) | 377 (4.5) |  |
|  | 25 to 34 years | 18,711 (16.0) | 17,736 (16.3) | 975 (11.6) |  |
|  | 35 to 49 years | 33,509 (28.6) | 30,209 (27.8) | 3,300 (39.1) |  |
|  | 50 to 64 years | 53,635 (45.8) | 49,862 (45.9) | 3,773 (44.7) |  |
| Sex (*n*, %) | Male | 54,769 (46.8) | 51,659 (47.6) | 3,110 (36.8) | 21.9 |
|  | Female | 62,219 (53.2) | 56,889 (52.4) | 5,330 (63.2) |  |
| Ethnic group (*n*, %) | White | 106,958 (91.4) | 99,084 (91.3) | 7,874 (93.3) | 7.5 |
|  | Non-white | 10,030 (8.6) | 9,464 (8.7) | 566 (6.7) |  |
| Country/region of residence (*n*, %) | North East England | 3,943 (3.4) | 3,554 (3.3) | 389 (4.6) | 17.7 |
|  | North West England | 12,127 (10.4) | 11,065 (10.2) | 1,062 (12.6) |  |
|  | Yorkshire and the Humber | 8,789 (7.5) | 8,050 (7.4) | 739 (8.8) |  |
|  | East Midlands | 7,495 (6.4) | 6,899 (6.4) | 596 (7.1) |  |
|  | West Midlands | 8,553 (7.3) | 7,880 (7.3) | 673 (8.0) |  |
|  | East of England | 10,847 (9.3) | 10,045 (9.3) | 802 (9.5) |  |
|  | London | 21,420 (18.3) | 20,196 (18.6) | 1,224 (14.5) |  |
|  | South East England | 14,553 (12.4) | 13,622 (12.5) | 931 (11.0) |  |
|  | South West England | 9,264 (7.9) | 8,658 (8.0) | 606 (7.2) |  |
|  | Scotland | 10,266 (8.8) | 9,588 (8.8) | 678 (8.0) |  |
|  | Wales | 6,427 (5.5) | 5,972 (5.5) | 455 (5.4) |  |
|  | Northern Ireland | 3,304 (2.8) | 3,019 (2.8) | 285 (3.4) |  |
| Area deprivation quintile group (*n*, %) | 1 (most deprived) | 14,449 (12.4) | 13,189 (12.2) | 1,260 (14.9) | 7.2 |
|  | 2 | 21,078 (18.0) | 19,457 (17.9) | 1,621 (19.2) |  |
|  | 3 | 25,228 (21.6) | 23,465 (21.6) | 1,763 (20.9) |  |
|  | 4 | 27,163 (23.2) | 25,330 (23.3) | 1,833 (21.7) |  |
|  | 5 (least deprived) | 29,070 (24.8) | 27,107 (25.0) | 1,963 (23.3) |  |
| Self-reported health/disability status (*n*, %) | No long-term health conditions | 92,758 (79.3) | 86,360 (79.6) | 6,398 (75.8) | 8.6 |
|  | Health conditions without impact to day-to-day activities | 11,539 (9.9) | 10,603 (9.8) | 936 (11.1) |  |
|  | Day-to-day activities limited a little by health conditions | 7,131 (6.1) | 6,465 (6.0) | 666 (7.9) |  |
|  | Day-to-day activities limited a lot by health conditions | 5,560 (4.8) | 5,120 (4.7) | 440 (5.2) |  |

**Supplementary Table 2 *(continued)***

| **Characteristic** | **Level** | **All participants (*n*=116,988)** | **Never infected (*n*=108,548)** | **Ever reported Long Covid (*n*=8,440)** | **Absolute standardized difference (%)** |
| --- | --- | --- | --- | --- | --- |
| Employment status (*n*, %) | Employed | 85,271 (72.9) | 78,506 (72.3) | 6,765 (80.2) | 23.9 |
|  | Unemployed | 3,325 (2.8) | 3,146 (2.9) | 179 (2.1) |  |
|  | Not working and not looking for work | 10,637 (9.1) | 9,817 (9.0) | 820 (9.7) |  |
|  | Retired | 12,984 (11.1) | 12,446 (11.5) | 538 (6.4) |  |
|  | Student | 4,771 (4.1) | 4,633 (4.3) | 138 (1.6) |  |
| Employment sector, among participants in employment (*n*, %) | Teaching and education | 8,876 (10.4) | 7,791 (9.9) | 1,085 (16.0) | 18.0 |
|  | Health care | 7,348 (8.6) | 6,709 (8.5) | 639 (9.4) |  |
|  | Social care | 2,152 (2.5) | 1,946 (2.5) | 206 (3.0) |  |
|  | Transport | 2,916 (3.4) | 2,694 (3.4) | 222 (3.3) |  |
|  | Retail and wholesale | 5,826 (6.8) | 5,415 (6.9) | 411 (6.1) |  |
|  | Hospitality | 2,606 (3.1) | 2,412 (3.1) | 194 (2.9) |  |
|  | Food production, agriculture and farming | 1,428 (1.7) | 1,331 (1.7) | 97 (1.4) |  |
|  | Personal services | 1,043 (1.2) | 973 (1.2) | 70 (1.0) |  |
|  | Information technology and communication | 5,808 (6.8) | 5,506 (7.0) | 302 (4.5) |  |
|  | Financial services | 6,156 (7.2) | 5,791 (7.4) | 365 (5.4) |  |
|  | Manufacturing and construction | 7,717 (9.0) | 7,152 (9.1) | 565 (8.4) |  |
|  | Civil service and local government | 5,403 (6.3) | 4,912 (6.3) | 491 (7.3) |  |
|  | Armed forces | 297 (0.3) | 279 (0.4) | 18 (0.3) |  |
|  | Arts, entertainment and recreation | 2,114 (2.5) | 1,994 (2.5) | 120 (1.8) |  |
|  | Other | 11,794 (13.8) | 10,976 (14.0) | 818 (12.1) |  |
|  | Unknown | 13,787 (16.2) | 12,625 (16.1) | 1,162 (17.2) |  |
| SOC Major Group, among participants in employment (*n*, %) | Managers, directors and senior officials | 7,406 (8.7) | 6,872 (8.8) | 534 (7.9) | 19.1 |
|  | Professional occupations | 18,709 (21.9) | 17,331 (22.1) | 1,378 (20.4) |  |
|  | Associate professional and technical occupations | 13,770 (16.1) | 12,791 (16.3) | 979 (14.5) |  |
|  | Administrative and secretarial occupations | 9,802 (11.5) | 8,992 (11.5) | 810 (12.0) |  |
|  | Skilled trades occupations | 6,117 (7.2) | 5,680 (7.2) | 437 (6.5) |  |
|  | Caring, leisure and other service occupations | 5,375 (6.3) | 4,681 (6.0) | 694 (10.3) |  |
|  | Sales and customer service occupations | 4,296 (5.0) | 3,981 (5.1) | 315 (4.7) |  |
|  | Process, plant and machine operatives | 3,040 (3.6) | 2,826 (3.6) | 214 (3.2) |  |
|  | Elementary occupations | 4,116 (4.8) | 3,790 (4.8) | 326 (4.8) |  |
|  | Unknown | 12,640 (14.8) | 11,562 (14.7) | 1,078 (15.9) |  |
| Self-employment status, among participants in employment (*n*, %) | Employee | 76,980 (90.3) | 70,776 (90.2) | 6,204 (91.7) | 5.4 |
|  | Self-employed | 8,291 (9.7) | 7,730 (9.8) | 561 (8.3) |  |

Notes: SD: standard deviation; SOC: Standard Occupational Classification. Area deprivation was based on the English Indices of Deprivation 2019, the Welsh Index of Multiple Deprivation 2019, the Scottish Index of Multiple Deprivation 2020, and the Northern Ireland Multiple Deprivation Measure 2017. Health conditions were self-reported rather than clinically diagnosed based on the survey question: “Do you have any physical or mental health conditions or illnesses lasting or expected to last 12 months or more (excluding any long-lasting COVID-19 symptoms)?”

**Supplementary Table 3.** Adjusted odds ratios for inactivity (excluding retirement) for participants currently reporting Long Covid compared with the pre-infection period, by time since SARS-CoV-2 infection and effect modifiers

| **Effect modifier** | **Level** | **Time since infection** | **aOR (95% CI)** | **Uncorrected p-value** | **Corrected p-value** |
| --- | --- | --- | --- | --- | --- |
| Age group (6 tests) | 16 to 49 years | 12 to <18 weeks | 0.73 (0.54 to 0.98) | Ref | Ref |
|  |  | 18 to <24 weeks | 0.93 (0.67 to 1.29) | Ref | Ref |
|  |  | 24 to <30 weeks | 0.98 (0.69 to 1.39) | Ref | Ref |
|  |  | 30 to <40 weeks | 1.18 (0.85 to 1.64) | Ref | Ref |
|  |  | 40 to <52 weeks | 0.99 (0.68 to 1.45) | Ref | Ref |
|  |  | ≥52 weeks | 1.01 (0.70 to 1.46) | Ref | Ref |
|  | 50 to 64 years | 12 to <18 weeks | 0.92 (0.71 to 1.18) | 0.24 | 0.59 |
|  |  | 18 to <24 weeks | 1.29 (0.97 to 1.72) | 0.13 | 0.49 |
|  |  | 24 to <30 weeks | 1.43 (1.04 to 1.95) | 0.11 | 0.49 |
|  |  | 30 to <40 weeks | 1.71 (1.28 to 2.29) | 0.09 | 0.49 |
|  |  | 40 to <52 weeks | 1.70 (1.22 to 2.37) | 0.03 | 0.49 |
|  |  | ≥52 weeks | 1.37 (1.02 to 1.83) | 0.21 | 0.59 |
| Sex (6 tests) | Male | 12 to <18 weeks | 0.99 (0.68 to 1.45) | Ref | Ref |
|  |  | 18 to <24 weeks | 0.90 (0.58 to 1.39) | Ref | Ref |
|  |  | 24 to <30 weeks | 1.03 (0.67 to 1.59) | Ref | Ref |
|  |  | 30 to <40 weeks | 1.57 (1.05 to 2.35) | Ref | Ref |
|  |  | 40 to <52 weeks | 1.41 (0.89 to 2.24) | Ref | Ref |
|  |  | ≥52 weeks | 0.80 (0.52 to 1.21) | Ref | Ref |
|  | Female | 12 to <18 weeks | 0.77 (0.62 to 0.96) | 0.25 | >0.99 |
|  |  | 18 to <24 weeks | 1.19 (0.93 to 1.54) | 0.27 | >0.99 |
|  |  | 24 to <30 weeks | 1.28 (0.97 to 1.69) | 0.41 | >0.99 |
|  |  | 30 to <40 weeks | 1.39 (1.08 to 1.81) | 0.63 | >0.99 |
|  |  | 40 to <52 weeks | 1.30 (0.97 to 1.75) | 0.77 | >0.99 |
|  |  | ≥52 weeks | 1.41 (1.07 to 1.86) | 0.03 | 0.37 |
| Ethnic group (6 tests) | Non-white | 12 to <18 weeks | 0.57 (0.27 to 1.20) | Ref | Ref |
|  |  | 18 to <24 weeks | 0.64 (0.28 to 1.49) | Ref | Ref |
|  |  | 24 to <30 weeks | 0.68 (0.28 to 1.69) | Ref | Ref |
|  |  | 30 to <40 weeks | 0.66 (0.28 to 1.57) | Ref | Ref |
|  |  | 40 to <52 weeks | 0.59 (0.25 to 1.42) | Ref | Ref |
|  |  | ≥52 weeks | 1.15 (0.55 to 2.41) | Ref | Ref |
|  | White | 12 to <18 weeks | 0.84 (0.69 to 1.03) | 0.32 | 0.95 |
|  |  | 18 to <24 weeks | 1.15 (0.92 to 1.44) | 0.19 | 0.77 |
|  |  | 24 to <30 weeks | 1.24 (0.98 to 1.58) | 0.21 | 0.77 |
|  |  | 30 to <40 weeks | 1.51 (1.21 to 1.90) | 0.07 | 0.51 |
|  |  | 40 to <52 weeks | 1.42 (1.10 to 1.85) | 0.06 | 0.51 |
|  |  | ≥52 weeks | 1.18 (0.93 to 1.51) | 0.95 | >0.99 |

**Supplementary Table 3 *(continued)***

| **Effect modifier** | **Level** | **Time since infection** | **aOR (95% CI)** | **Uncorrected p-value** | **Corrected p-value** |
| --- | --- | --- | --- | --- | --- |
| Area deprivation quintile group (24 tests) | 1 (most deprived) | 12 to <18 weeks | 0.69 (0.47 to 1.02) | Ref | Ref |
|  |  | 18 to <24 weeks | 1.05 (0.68 to 1.62) | Ref | Ref |
|  |  | 24 to <30 weeks | 1.35 (0.83 to 2.19) | Ref | Ref |
|  |  | 30 to <40 weeks | 1.16 (0.76 to 1.78) | Ref | Ref |
|  |  | 40 to <52 weeks | 1.17 (0.72 to 1.90) | Ref | Ref |
|  |  | ≥52 weeks | 1.05 (0.69 to 1.59) | Ref | Ref |
|  | 2 | 12 to <18 weeks | 1.15 (0.77 to 1.71) | 0.07 | >0.99 |
|  |  | 18 to <24 weeks | 1.27 (0.83 to 1.96) | 0.53 | >0.99 |
|  |  | 24 to <30 weeks | 1.22 (0.75 to 1.98) | 0.78 | >0.99 |
|  |  | 30 to <40 weeks | 2.23 (1.44 to 3.47) | 0.04 | >0.99 |
|  |  | 40 to <52 weeks | 2.01 (1.23 to 3.28) | 0.13 | >0.99 |
|  |  | ≥52 weeks | 2.09 (1.31 to 3.32) | 0.03 | >0.99 |
|  | 3 | 12 to <18 weeks | 0.97 (0.61 to 1.55) | 0.27 | >0.99 |
|  |  | 18 to <24 weeks | 0.84 (0.49 to 1.46) | 0.54 | >0.99 |
|  |  | 24 to <30 weeks | 1.16 (0.66 to 2.03) | 0.69 | >0.99 |
|  |  | 30 to <40 weeks | 1.21 (0.70 to 2.10) | 0.91 | >0.99 |
|  |  | 40 to <52 weeks | 1.76 (0.92 to 3.36) | 0.32 | >0.99 |
|  |  | ≥52 weeks | 0.81 (0.45 to 1.46) | 0.48 | >0.99 |
|  | 4 | 12 to <18 weeks | 0.73 (0.47 to 1.13) | 0.88 | >0.99 |
|  |  | 18 to <24 weeks | 1.47 (0.88 to 2.47) | 0.32 | >0.99 |
|  |  | 24 to <30 weeks | 1.06 (0.61 to 1.84) | 0.52 | >0.99 |
|  |  | 30 to <40 weeks | 1.45 (0.87 to 2.42) | 0.52 | >0.99 |
|  |  | 40 to <52 weeks | 1.21 (0.65 to 2.23) | 0.94 | >0.99 |
|  |  | ≥52 weeks | 1.29 (0.72 to 2.32) | 0.57 | >0.99 |
|  | 5 (least deprived) | 12 to <18 weeks | 0.69 (0.42 to 1.12) | 0.97 | >0.99 |
|  |  | 18 to <24 weeks | 1.06 (0.61 to 1.84) | 0.97 | >0.99 |
|  |  | 24 to <30 weeks | 1.25 (0.72 to 2.20) | 0.85 | >0.99 |
|  |  | 30 to <40 weeks | 1.40 (0.81 to 2.44) | 0.60 | >0.99 |
|  |  | 40 to <52 weeks | 0.81 (0.41 to 1.57) | 0.37 | >0.99 |
|  |  | ≥52 weeks | 0.87 (0.44 to 1.70) | 0.64 | >0.99 |

**Supplementary Table 3 *(continued)***

| **Effect modifier** | **Level** | **Time since infection** | **aOR (95% CI)** | **Uncorrected p-value** | **Corrected p-value** |
| --- | --- | --- | --- | --- | --- |
| Self-reported health/disability status (18 tests) | No long-term health conditions | 12 to <18 weeks | 0.71 (0.55 to 0.91) | Ref | Ref |
|  |  | 18 to <24 weeks | 1.13 (0.85 to 1.49) | Ref | Ref |
|  |  | 24 to <30 weeks | 0.98 (0.72 to 1.33) | Ref | Ref |
|  |  | 30 to <40 weeks | 1.42 (1.07 to 1.89) | Ref | Ref |
|  |  | 40 to <52 weeks | 1.09 (0.79 to 1.50) | Ref | Ref |
|  |  | ≥52 weeks | 1.13 (0.84 to 1.53) | Ref | Ref |
|  | Health conditions without impact to day-to-day activities | 12 to <18 weeks | 0.77 (0.42 to 1.44) | 0.79 | >0.99 |
|  |  | 18 to <24 weeks | 1.05 (0.53 to 2.09) | 0.86 | >0.99 |
|  |  | 24 to <30 weeks | 1.34 (0.66 to 2.73) | 0.42 | >0.99 |
|  |  | 30 to <40 weeks | 1.53 (0.78 to 3.00) | 0.84 | >0.99 |
|  |  | 40 to <52 weeks | 2.56 (1.34 to 4.91) | 0.02 | 0.66 |
|  |  | ≥52 weeks | 1.49 (0.77 to 2.87) | 0.45 | >0.99 |
|  | Day-to-day activities limited a little by health conditions | 12 to <18 weeks | 1.08 (0.66 to 1.78) | 0.14 | >0.99 |
|  |  | 18 to <24 weeks | 1.04 (0.59 to 1.84) | 0.81 | >0.99 |
|  |  | 24 to <30 weeks | 2.35 (1.20 to 4.63) | 0.02 | 0.66 |
|  |  | 30 to <40 weeks | 1.73 (0.91 to 3.26) | 0.59 | >0.99 |
|  |  | 40 to <52 weeks | 1.63 (0.78 to 3.41) | 0.33 | >0.99 |
|  |  | ≥52 weeks | 2.07 (1.09 to 3.93) | 0.10 | >0.99 |
|  | Day-to-day activities limited a lot by health conditions | 12 to <18 weeks | 1.15 (0.71 to 1.87) | 0.08 | >0.99 |
|  |  | 18 to <24 weeks | 1.24 (0.70 to 2.20) | 0.77 | >0.99 |
|  |  | 24 to <30 weeks | 1.38 (0.77 to 2.48) | 0.31 | >0.99 |
|  |  | 30 to <40 weeks | 1.35 (0.80 to 2.28) | 0.87 | >0.99 |
|  |  | 40 to <52 weeks | 1.42 (0.72 to 2.77) | 0.49 | >0.99 |
|  |  | ≥52 weeks | 0.72 (0.40 to 1.31) | 0.19 | >0.99 |
| Reinfected with SARS-CoV-2 (6 tests) | No | 12 to <18 weeks | 0.81 (0.67 to 0.98) | Ref | Ref |
|  |  | 18 to <24 weeks | 1.11 (0.89 to 1.39) | Ref | Ref |
|  |  | 24 to <30 weeks | 1.16 (0.91 to 1.47) | Ref | Ref |
|  |  | 30 to <40 weeks | 1.49 (1.18 to 1.87) | Ref | Ref |
|  |  | 40 to <52 weeks | 1.37 (1.04 to 1.81) | Ref | Ref |
|  |  | ≥52 weeks | 1.13 (0.86 to 1.47) | Ref | Ref |
|  | Yes | 12 to <18 weeks | 3.47 (0.77 to 15.64) | 0.06 | 0.83 |
|  |  | 18 to <24 weeks | 1.46 (0.55 to 3.88) | 0.59 | >0.99 |
|  |  | 24 to <30 weeks | 2.54 (0.98 to 6.62) | 0.12 | 0.83 |
|  |  | 30 to <40 weeks | 1.49 (0.72 to 3.07) | >0.99 | >0.99 |
|  |  | 40 to <52 weeks | 1.47 (0.75 to 2.88) | 0.85 | >0.99 |
|  |  | ≥52 weeks | 1.69 (0.99 to 2.90) | 0.17 | 0.83 |

**Supplementary Table 3 *(continued)***

| **Effect modifier** | **Level** | **Time since infection** | **aOR (95% CI)** | **Uncorrected p-value** | **Corrected p-value** |
| --- | --- | --- | --- | --- | --- |
| Mode of data collection (6 tests) | Face-to-face | 12 to <18 weeks | 0.88 (0.72 to 1.08) | Ref | Ref |
|  |  | 18 to <24 weeks | 1.15 (0.90 to 1.46) | Ref | Ref |
|  |  | 24 to <30 weeks | 1.16 (0.89 to 1.52) | Ref | Ref |
|  |  | 30 to <40 weeks | 1.21 (0.93 to 1.56) | Ref | Ref |
|  |  | 40 to <52 weeks | 1.31 (0.97 to 1.77) | Ref | Ref |
|  |  | ≥52 weeks | 1.11 (0.84 to 1.46) | Ref | Ref |
|  | Remote | 12 to <18 weeks | 0.58 (0.31 to 1.07) | 0.20 | >0.99 |
|  |  | 18 to <24 weeks | 1.02 (0.62 to 1.69) | 0.69 | >0.99 |
|  |  | 24 to <30 weeks | 1.30 (0.79 to 2.12) | 0.70 | >0.99 |
|  |  | 30 to <40 weeks | 2.21 (1.46 to 3.35) | 0.01 | 0.22 |
|  |  | 40 to <52 weeks | 1.20 (0.76 to 1.87) | 0.73 | >0.99 |
|  |  | ≥52 weeks | 1.27 (0.90 to 1.79) | 0.52 | >0.99 |

Notes: aOR: adjusted odds ratio; CI: confidence interval; Ref: reference category. Estimates are from conditional logit models including the exposure variable interacted with each of the effect modifiers, adjusted for calendar day of study assessment, current age, and interactions between calendar day and each of current age, sex, self-reported health/disability status at survey enrolment, and each of the effect modifiers (excluding reinfection status and data collection mode). P-values were corrected using the Benjamini-Yekutieli method.

**Supplementary Table 4.** Adjusted odds ratios for long-term (≥4 weeks) absence for participants currently reporting Long Covid compared with the pre-infection period, by time since SARS-CoV-2 infection and effect modifiers

| **Effect modifier** | **Level** | **Time since infection** | **aOR (95% CI)** | **Uncorrected p-value** | **Corrected p-value** |
| --- | --- | --- | --- | --- | --- |
| Age group (6 tests) | 16 to 49 years | 12 to <18 weeks | 0.99 (0.65 to 1.52) | Ref | Ref |
|  |  | 18 to <24 weeks | 1.36 (0.89 to 2.09) | Ref | Ref |
|  |  | 24 to <30 weeks | 1.05 (0.64 to 1.70) | Ref | Ref |
|  |  | 30 to <40 weeks | 0.85 (0.52 to 1.40) | Ref | Ref |
|  |  | 40 to <52 weeks | 0.84 (0.48 to 1.48) | Ref | Ref |
|  |  | ≥52 weeks | 0.51 (0.27 to 0.97) | Ref | Ref |
|  | 50 to 64 years | 12 to <18 weeks | 1.46 (0.97 to 2.20) | 0.20 | 0.83 |
|  |  | 18 to <24 weeks | 1.48 (0.97 to 2.28) | 0.78 | >0.99 |
|  |  | 24 to <30 weeks | 2.03 (1.27 to 3.26) | 0.053 | 0.39 |
|  |  | 30 to <40 weeks | 1.28 (0.82 to 2.01) | 0.23 | 0.83 |
|  |  | 40 to <52 weeks | 1.00 (0.61 to 1.66) | 0.64 | >0.99 |
|  |  | ≥52 weeks | 1.26 (0.73 to 2.18) | 0.03 | 0.39 |
| Sex (6 tests) | Male | 12 to <18 weeks | 1.32 (0.79 to 2.23) | Ref | Ref |
|  |  | 18 to <24 weeks | 1.33 (0.77 to 2.31) | Ref | Ref |
|  |  | 24 to <30 weeks | 1.88 (1.05 to 3.39) | Ref | Ref |
|  |  | 30 to <40 weeks | 1.13 (0.62 to 2.09) | Ref | Ref |
|  |  | 40 to <52 weeks | 0.93 (0.46 to 1.89) | Ref | Ref |
|  |  | ≥52 weeks | 0.67 (0.31 to 1.44) | Ref | Ref |
|  | Female | 12 to <18 weeks | 1.15 (0.81 to 1.65) | 0.67 | >0.99 |
|  |  | 18 to <24 weeks | 1.45 (1.01 to 2.09) | 0.80 | >0.99 |
|  |  | 24 to <30 weeks | 1.28 (0.85 to 1.95) | 0.30 | >0.99 |
|  |  | 30 to <40 weeks | 1.00 (0.67 to 1.48) | 0.73 | >0.99 |
|  |  | 40 to <52 weeks | 0.86 (0.55 to 1.35) | 0.84 | >0.99 |
|  |  | ≥52 weeks | 0.92 (0.56 to 1.53) | 0.49 | >0.99 |
| Ethnic group (6 tests) | Non-white | 12 to <18 weeks | 1.26 (0.31 to 5.06) | Ref | Ref |
|  |  | 18 to <24 weeks | 1.08 (0.24 to 4.91) | Ref | Ref |
|  |  | 24 to <30 weeks | 0.51 (0.06 to 4.23) | Ref | Ref |
|  |  | 30 to <40 weeks | 0.82 (0.19 to 3.57) | Ref | Ref |
|  |  | 40 to <52 weeks | 0.32 (0.05 to 1.98) | Ref | Ref |
|  |  | ≥52 weeks | 0.27 (0.05 to 1.40) | Ref | Ref |
|  | White | 12 to <18 weeks | 1.20 (0.89 to 1.63) | 0.95 | >0.99 |
|  |  | 18 to <24 weeks | 1.41 (1.04 to 1.93) | 0.74 | >0.99 |
|  |  | 24 to <30 weeks | 1.52 (1.07 to 2.14) | 0.32 | >0.99 |
|  |  | 30 to <40 weeks | 1.05 (0.75 to 1.48) | 0.75 | >0.99 |
|  |  | 40 to <52 weeks | 0.93 (0.63 to 1.38) | 0.26 | >0.99 |
|  |  | ≥52 weeks | 0.89 (0.58 to 1.38) | 0.17 | >0.99 |

**Supplementary Table 4 *(continued)***

| **Effect modifier** | **Level** | **Time since infection** | **aOR (95% CI)** | **Uncorrected p-value** | **Corrected p-value** |
| --- | --- | --- | --- | --- | --- |
| Area deprivation quintile group (24 tests) | 1 (most deprived) | 12 to <18 weeks | 0.71 (0.33 to 1.50) | Ref | Ref |
|  |  | 18 to <24 weeks | 0.41 (0.16 to 1.08) | Ref | Ref |
|  |  | 24 to <30 weeks | 0.85 (0.35 to 2.05) | Ref | Ref |
|  |  | 30 to <40 weeks | 0.72 (0.29 to 1.80) | Ref | Ref |
|  |  | 40 to <52 weeks | 0.73 (0.29 to 1.84) | Ref | Ref |
|  |  | ≥52 weeks | 0.43 (0.15 to 1.20) | Ref | Ref |
|  | 2 | 12 to <18 weeks | 1.29 (0.66 to 2.50) | 0.24 | >0.99 |
|  |  | 18 to <24 weeks | 2.26 (1.25 to 4.09) | 0.003 | 0.26 |
|  |  | 24 to <30 weeks | 1.63 (0.81 to 3.28) | 0.25 | >0.99 |
|  |  | 30 to <40 weeks | 1.10 (0.53 to 2.27) | 0.49 | >0.99 |
|  |  | 40 to <52 weeks | 1.30 (0.56 to 2.98) | 0.37 | >0.99 |
|  |  | ≥52 weeks | 1.58 (0.67 to 3.73) | 0.06 | >0.99 |
|  | 3 | 12 to <18 weeks | 1.73 (0.91 to 3.32) | 0.08 | >0.99 |
|  |  | 18 to <24 weeks | 1.54 (0.78 to 3.03) | 0.03 | 0.85 |
|  |  | 24 to <30 weeks | 1.87 (0.86 to 4.02) | 0.19 | >0.99 |
|  |  | 30 to <40 weeks | 1.32 (0.64 to 2.76) | 0.31 | >0.99 |
|  |  | 40 to <52 weeks | 0.74 (0.30 to 1.84) | 0.99 | >0.99 |
|  |  | ≥52 weeks | 0.33 (0.11 to 1.06) | 0.75 | >0.99 |
|  | 4 | 12 to <18 weeks | 1.55 (0.81 to 2.99) | 0.12 | >0.99 |
|  |  | 18 to <24 weeks | 2.13 (1.09 to 4.16) | 0.01 | 0.26 |
|  |  | 24 to <30 weeks | 2.34 (1.15 to 4.75) | 0.08 | >0.99 |
|  |  | 30 to <40 weeks | 1.18 (0.58 to 2.42) | 0.40 | >0.99 |
|  |  | 40 to <52 weeks | 0.83 (0.37 to 1.87) | 0.85 | >0.99 |
|  |  | ≥52 weeks | 0.80 (0.33 to 1.98) | 0.37 | >0.99 |
|  | 5 (least deprived) | 12 to <18 weeks | 0.96 (0.51 to 1.81) | 0.54 | >0.99 |
|  |  | 18 to <24 weeks | 1.09 (0.55 to 2.13) | 0.11 | >0.99 |
|  |  | 24 to <30 weeks | 0.95 (0.42 to 2.14) | 0.85 | >0.99 |
|  |  | 30 to <40 weeks | 0.88 (0.44 to 1.79) | 0.73 | >0.99 |
|  |  | 40 to <52 weeks | 1.04 (0.45 to 2.39) | 0.58 | >0.99 |
|  |  | ≥52 weeks | 1.45 (0.56 to 3.74) | 0.09 | >0.99 |

**Supplementary Table 4 *(continued)***

| **Effect modifier** | **Level** | **Time since infection** | **aOR (95% CI)** | **Uncorrected p-value** | **Corrected p-value** |
| --- | --- | --- | --- | --- | --- |
| Presence of self-reported health conditions (6 tests) | No | 12 to <18 weeks | 1.26 (0.89 to 1.79) | Ref | Ref |
|  |  | 18 to <24 weeks | 1.96 (1.38 to 2.78) | Ref | Ref |
|  |  | 24 to <30 weeks | 1.42 (0.95 to 2.12) | Ref | Ref |
|  |  | 30 to <40 weeks | 1.00 (0.67 to 1.49) | Ref | Ref |
|  |  | 40 to <52 weeks | 0.97 (0.62 to 1.53) | Ref | Ref |
|  |  | ≥52 weeks | 1.05 (0.65 to 1.71) | Ref | Ref |
|  | Yes | 12 to <18 weeks | 1.06 (0.61 to 1.85) | 0.61 | >0.99 |
|  |  | 18 to <24 weeks | 0.58 (0.31 to 1.10) | 0.001 | 0.02 |
|  |  | 24 to <30 weeks | 1.51 (0.80 to 2.86) | 0.87 | >0.99 |
|  |  | 30 to <40 weeks | 1.04 (0.57 to 1.90) | 0.91 | >0.99 |
|  |  | 40 to <52 weeks | 0.67 (0.33 to 1.37) | 0.40 | >0.99 |
|  |  | ≥52 weeks | 0.45 (0.20 to 1.01) | 0.08 | 0.57 |
| Reinfected with SARS-CoV-2 (6 tests) | No | 12 to <18 weeks | 1.17 (0.87 to 1.58) | Ref | Ref |
|  |  | 18 to <24 weeks | 1.35 (0.98 to 1.85) | Ref | Ref |
|  |  | 24 to <30 weeks | 1.55 (1.08 to 2.22) | Ref | Ref |
|  |  | 30 to <40 weeks | 0.93 (0.65 to 1.34) | Ref | Ref |
|  |  | 40 to <52 weeks | 0.85 (0.56 to 1.30) | Ref | Ref |
|  |  | ≥52 weeks | 0.69 (0.42 to 1.11) | Ref | Ref |
|  | Yes | 12 to <18 weeks | 4.46 (0.51 to 39.38) | 0.23 | >0.99 |
|  |  | 18 to <24 weeks | 1.93 (0.64 to 5.83) | 0.53 | >0.99 |
|  |  | 24 to <30 weeks | 0.74 (0.24 to 2.28) | 0.22 | >0.99 |
|  |  | 30 to <40 weeks | 1.33 (0.55 to 3.22) | 0.46 | >0.99 |
|  |  | 40 to <52 weeks | 0.71 (0.28 to 1.84) | 0.73 | >0.99 |
|  |  | ≥52 weeks | 0.96 (0.44 to 2.10) | 0.40 | >0.99 |
| Mode of data collection (6 tests) | Face-to-face | 12 to <18 weeks | 1.00 (0.71 to 1.41) | Ref | Ref |
|  |  | 18 to <24 weeks | 1.01 (0.69 to 1.49) | Ref | Ref |
|  |  | 24 to <30 weeks | 1.15 (0.72 to 1.81) | Ref | Ref |
|  |  | 30 to <40 weeks | 0.89 (0.56 to 1.41) | Ref | Ref |
|  |  | 40 to <52 weeks | 0.95 (0.57 to 1.59) | Ref | Ref |
|  |  | ≥52 weeks | 0.72 (0.42 to 1.23) | Ref | Ref |
|  | Remote | 12 to <18 weeks | 2.19 (1.14 to 4.21) | 0.04 | 0.19 |
|  |  | 18 to <24 weeks | 2.37 (1.41 to 3.99) | 0.01 | 0.13 |
|  |  | 24 to <30 weeks | 1.79 (1.07 to 2.98) | 0.20 | 0.73 |
|  |  | 30 to <40 weeks | 1.05 (0.66 to 1.69) | 0.61 | >0.99 |
|  |  | 40 to <52 weeks | 0.44 (0.25 to 0.79) | 0.04 | 0.19 |
|  |  | ≥52 weeks | 0.63 (0.37 to 1.07) | 0.59 | >0.99 |

**Supplementary Table 4 *(continued)***

| **Effect modifier** | **Level** | **Time since infection** | **aOR (95% CI)** | **Uncorrected p-value** | **Corrected p-value** |
| --- | --- | --- | --- | --- | --- |
| Employment sector, among participants in employment (42 tests) | Teaching and education | 12 to <18 weeks | 0.89 (0.43 to 1.81) | Ref | Ref |
|  |  | 18 to <24 weeks | 1.12 (0.59 to 2.11) | Ref | Ref |
|  |  | 24 to <30 weeks | 2.06 (1.08 to 3.96) | Ref | Ref |
|  |  | 30 to <40 weeks | 1.00 (0.52 to 1.90) | Ref | Ref |
|  |  | 40 to <52 weeks | 0.69 (0.31 to 1.56) | Ref | Ref |
|  |  | ≥52 weeks | 1.39 (0.56 to 3.44) | Ref | Ref |
|  | Health or social care | 12 to <18 weeks | 2.07 (1.15 to 3.73) | 0.07 | >0.99 |
|  |  | 18 to <24 weeks | 2.00 (1.04 to 3.84) | 0.21 | >0.99 |
|  |  | 24 to <30 weeks | 0.99 (0.42 to 2.31) | 0.18 | >0.99 |
|  |  | 30 to <40 weeks | 0.82 (0.38 to 1.78) | 0.71 | >0.99 |
|  |  | 40 to <52 weeks | 0.85 (0.35 to 2.04) | 0.74 | >0.99 |
|  |  | ≥52 weeks | 0.46 (0.16 to 1.31) | 0.12 | >0.99 |
|  | Transport | 12 to <18 weeks | 0.23 (0.02 to 2.19) | 0.26 | >0.99 |
|  |  | 18 to <24 weeks | 0.77 (0.11 to 5.17) | 0.72 | >0.99 |
|  |  | 24 to <30 weeks | 0.53 (0.10 to 2.70) | 0.13 | >0.99 |
|  |  | 30 to <40 weeks | 0.52 (0.10 to 2.71) | 0.47 | >0.99 |
|  |  | 40 to <52 weeks | 0.34 (0.05 to 2.34) | 0.51 | >0.99 |
|  |  | ≥52 weeks | 1.48 (0.25 to 8.68) | 0.95 | >0.99 |
|  | Retail and wholesale | 12 to <18 weeks | 1.30 (0.30 to 5.63) | 0.64 | >0.99 |
|  |  | 18 to <24 weeks | 0.49 (0.09 to 2.78) | 0.38 | >0.99 |
|  |  | 24 to <30 weeks | 2.05 (0.39 to 10.82) | 0.99 | >0.99 |
|  |  | 30 to <40 weeks | 0.96 (0.22 to 4.11) | 0.96 | >0.99 |
|  |  | 40 to <52 weeks | 0.97 (0.23 to 4.12) | 0.69 | >0.99 |
|  |  | ≥52 weeks | 0.74 (0.14 to 3.81) | 0.51 | >0.99 |
|  | Hospitality | 12 to <18 weeks | 0.65 (0.07 to 5.85) | 0.80 | >0.99 |
|  |  | 18 to <24 weeks | 3.35 (0.47 to 23.84) | 0.30 | >0.99 |
|  |  | 24 to <30 weeks | 6.69 (0.84 to 53.53) | 0.29 | >0.99 |
|  |  | 30 to <40 weeks | 2.98 (0.39 to 22.90) | 0.32 | >0.99 |
|  |  | 40 to <52 weeks | 6.99 (0.85 to 57.43) | 0.04 | >0.99 |
|  |  | ≥52 weeks | 3.97 (0.25 to 62.24) | 0.48 | >0.99 |
|  | Manufacturing and construction | 12 to <18 weeks | 1.30 (0.42 to 4.05) | 0.58 | >0.99 |
|  |  | 18 to <24 weeks | 2.06 (0.64 to 6.61) | 0.36 | >0.99 |
|  |  | 24 to <30 weeks | 2.84 (0.78 to 10.40) | 0.67 | >0.99 |
|  |  | 30 to <40 weeks | 1.26 (0.21 to 7.63) | 0.81 | >0.99 |
|  |  | 40 to <52 weeks | 2.22 (0.35 to 13.91) | 0.26 | >0.99 |
|  |  | ≥52 weeks | 1.12 (0.17 to 7.18) | 0.84 | >0.99 |

**Supplementary Table 4 *(continued)***

| **Effect modifier** | **Level** | **Time since infection** | **aOR (95% CI)** | **Uncorrected p-value** | **Corrected p-value** |
| --- | --- | --- | --- | --- | --- |
| Employment sector, among participants in employment (42 tests) *(continued)* | Civil service and local government | 12 to <18 weeks | 0.92 (0.32 to 2.68) | 0.95 | >0.99 |
|  |  | 18 to <24 weeks | 0.44 (0.12 to 1.55) | 0.19 | >0.99 |
|  |  | 24 to <30 weeks | 0.28 (0.05 to 1.47) | 0.03 | >0.99 |
|  |  | 30 to <40 weeks | 0.40 (0.10 to 1.51) | 0.22 | >0.99 |
|  |  | 40 to <52 weeks | 0.11 (0.02 to 0.62) | 0.06 | >0.99 |
|  |  | ≥52 weeks | 0.06 (0.01 to 0.37) | 0.002 | 0.41 |
|  | Other | 12 to <18 weeks | 0.72 (0.34 to 1.52) | 0.69 | >0.99 |
|  |  | 18 to <24 weeks | 1.53 (0.78 to 3.00) | 0.50 | >0.99 |
|  |  | 24 to <30 weeks | 1.17 (0.50 to 2.70) | 0.29 | >0.99 |
|  |  | 30 to <40 weeks | 0.93 (0.40 to 2.15) | 0.89 | >0.99 |
|  |  | 40 to <52 weeks | 0.23 (0.08 to 0.69) | 0.11 | >0.99 |
|  |  | ≥52 weeks | 0.27 (0.10 to 0.72) | 0.01 | >0.99 |
| SOC Major Group, among participants in employment (42 tests) | Managers, directors and senior officials | 12 to <18 weeks | 1.16 (0.32 to 4.19) | Ref | Ref |
|  |  | 18 to <24 weeks | 1.56 (0.32 to 7.65) | Ref | Ref |
|  |  | 24 to <30 weeks | 3.02 (0.79 to 11.45) | Ref | Ref |
|  |  | 30 to <40 weeks | 1.09 (0.20 to 5.91) | Ref | Ref |
|  |  | 40 to <52 weeks | 2.41 (0.47 to 12.30) | Ref | Ref |
|  |  | ≥52 weeks | 0.57 (0.07 to 4.67) | Ref | Ref |
|  | Professional occupations | 12 to <18 weeks | 1.35 (0.74 to 2.47) | 0.83 | >0.99 |
|  |  | 18 to <24 weeks | 1.68 (0.88 to 3.19) | 0.94 | >0.99 |
|  |  | 24 to <30 weeks | 1.35 (0.66 to 2.77) | 0.30 | >0.99 |
|  |  | 30 to <40 weeks | 1.12 (0.55 to 2.25) | 0.98 | >0.99 |
|  |  | 40 to <52 weeks | 0.87 (0.40 to 1.92) | 0.27 | >0.99 |
|  |  | ≥52 weeks | 0.84 (0.35 to 2.02) | 0.73 | >0.99 |
|  | Associate professional and technical occupations | 12 to <18 weeks | 1.31 (0.50 to 3.39) | 0.88 | >0.99 |
|  |  | 18 to <24 weeks | 1.72 (0.74 to 4.00) | 0.92 | >0.99 |
|  |  | 24 to <30 weeks | 1.58 (0.46 to 5.49) | 0.49 | >0.99 |
|  |  | 30 to <40 weeks | 1.73 (0.61 to 4.92) | 0.65 | >0.99 |
|  |  | 40 to <52 weeks | 0.74 (0.21 to 2.57) | 0.26 | >0.99 |
|  |  | ≥52 weeks | 0.70 (0.18 to 2.69) | 0.87 | >0.99 |
|  | Administrative and secretarial occupations | 12 to <18 weeks | 0.60 (0.18 to 2.05) | 0.47 | >0.99 |
|  |  | 18 to <24 weeks | 1.13 (0.41 to 3.08) | 0.73 | >0.99 |
|  |  | 24 to <30 weeks | 1.72 (0.50 to 5.85) | 0.54 | >0.99 |
|  |  | 30 to <40 weeks | 1.36 (0.46 to 4.04) | 0.83 | >0.99 |
|  |  | 40 to <52 weeks | 0.46 (0.11 to 1.86) | 0.13 | >0.99 |
|  |  | ≥52 weeks | 0.73 (0.20 to 2.60) | 0.84 | >0.99 |

**Supplementary Table 4 *(continued)***

| **Effect modifier** | **Level** | **Time since infection** | **aOR (95% CI)** | **Uncorrected p-value** | **Corrected p-value** |
| --- | --- | --- | --- | --- | --- |
| SOC Major Group, among participants in employment (42 tests) *(continued)* | Skilled trades occupations | 12 to <18 weeks | 0.12 (0.01 to 1.43) | 0.11 | >0.99 |
|  |  | 18 to <24 weeks | 0.67 (0.16 to 2.75) | 0.43 | >0.99 |
|  |  | 24 to <30 weeks | 3.69 (0.87 to 15.69) | 0.84 | >0.99 |
|  |  | 30 to <40 weeks | 0.45 (0.08 to 2.49) | 0.47 | >0.99 |
|  |  | 40 to <52 weeks | 1.70 (0.43 to 6.70) | 0.74 | >0.99 |
|  |  | ≥52 weeks | 1.13 (0.22 to 5.91) | 0.61 | >0.99 |
|  | Caring, leisure and other service occupations | 12 to <18 weeks | 1.67 (0.82 to 3.41) | 0.62 | >0.99 |
|  |  | 18 to <24 weeks | 1.28 (0.56 to 2.93) | 0.83 | >0.99 |
|  |  | 24 to <30 weeks | 1.76 (0.74 to 4.15) | 0.50 | >0.99 |
|  |  | 30 to <40 weeks | 1.55 (0.74 to 3.27) | 0.71 | >0.99 |
|  |  | 40 to <52 weeks | 0.70 (0.27 to 1.83) | 0.20 | >0.99 |
|  |  | ≥52 weeks | 0.59 (0.20 to 1.75) | 0.97 | >0.99 |
|  | Sales and customer service occupations | 12 to <18 weeks | 1.69 (0.33 to 8.79) | 0.72 | >0.99 |
|  |  | 18 to <24 weeks | 3.51 (0.49 to 25.18) | 0.53 | >0.99 |
|  |  | 24 to <30 weeks | <0.01 (<0.01 to >99.9) | 0.98 | >0.99 |
|  |  | 30 to <40 weeks | 1.67 (0.35 to 8.08) | 0.72 | >0.99 |
|  |  | 40 to <52 weeks | 0.48 (0.10 to 2.39) | 0.17 | >0.99 |
|  |  | ≥52 weeks | 0.47 (0.11 to 2.03) | 0.89 | >0.99 |
|  | Process, plant and machine operatives; and elementary occupations | 12 to <18 weeks | 1.22 (0.47 to 3.15) | 0.95 | >0.99 |
|  |  | 18 to <24 weeks | 1.91 (0.68 to 5.36) | 0.84 | >0.99 |
|  |  | 24 to <30 weeks | 2.12 (0.78 to 5.77) | 0.68 | >0.99 |
|  |  | 30 to <40 weeks | 1.34 (0.42 to 4.29) | 0.84 | >0.99 |
|  |  | 40 to <52 weeks | 1.24 (0.23 to 6.59) | 0.58 | >0.99 |
|  |  | ≥52 weeks | 1.22 (0.24 to 6.23) | 0.57 | >0.99 |
| Self-employment status, among participants in employment (6 tests) | Employee | 12 to <18 weeks | 1.21 (0.88 to 1.67) | Ref | Ref |
|  |  | 18 to <24 weeks | 1.39 (1.00 to 1.92) | Ref | Ref |
|  |  | 24 to <30 weeks | 1.35 (0.94 to 1.94) | Ref | Ref |
|  |  | 30 to <40 weeks | 1.14 (0.80 to 1.62) | Ref | Ref |
|  |  | 40 to <52 weeks | 0.75 (0.50 to 1.13) | Ref | Ref |
|  |  | ≥52 weeks | 0.71 (0.45 to 1.11) | Ref | Ref |
|  | Self-employed | 12 to <18 weeks | 1.04 (0.45 to 2.40) | 0.74 | >0.99 |
|  |  | 18 to <24 weeks | 1.23 (0.51 to 2.97) | 0.80 | >0.99 |
|  |  | 24 to <30 weeks | 1.35 (0.49 to 3.73) | >0.99 | >0.99 |
|  |  | 30 to <40 weeks | 0.18 (0.05 to 0.68) | 0.01 | 0.12 |
|  |  | 40 to <52 weeks | 1.38 (0.53 to 3.63) | 0.24 | >0.99 |
|  |  | ≥52 weeks | 0.79 (0.28 to 2.21) | 0.84 | >0.99 |

Notes: aOR: adjusted odds ratio; CI: confidence interval; Ref: reference category. Estimates are from conditional logit models including the exposure variable interacted with each of the effect modifiers, adjusted for calendar day of study assessment, current age, and interactions between calendar day and each of current age, sex, self-reported health/disability status at survey enrolment, and each of the effect modifiers (excluding reinfection status and data collection mode). Models were fitted to study assessments from 1 October 2021 when participants were in employment. P-values were corrected using the Benjamini-Yekutieli method.

**Supplementary Figure 1.** Study participant flow diagram for the analysis population


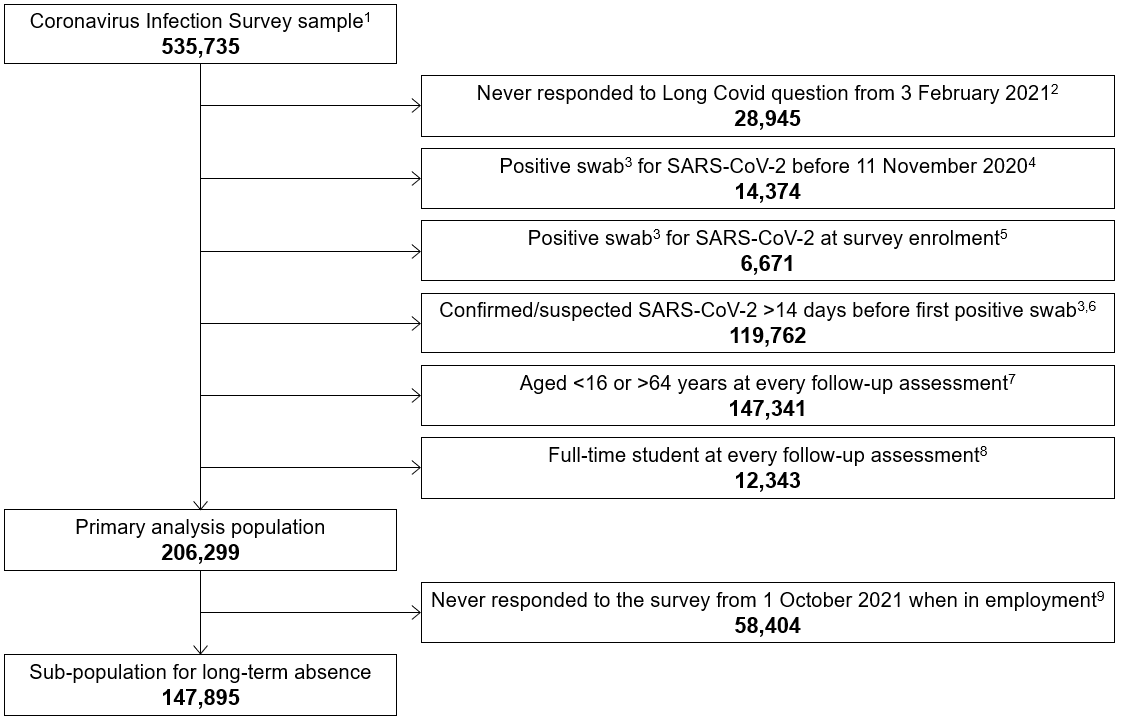


Notes:

1. 535,735 participants from 267,913 households enrolled into the Coronavirus Infection Survey (CIS). However, not all households who were invited to participate in the survey chose to enrol. Supplementary Table 1 reports household enrolment rates, which were as high as 51% at the start of the survey but dropped to as low as 12% by the end of recruitment.
2. The Long Covid survey question was introduced on the Coronavirus Infection Survey (CIS) on 3 February 2021.
3. Positive swab tests for SARS-CoV-2 included polymerase chain reaction (PCR) tests via CIS study assessments and all swabs taken outside of the study, as self-reported by participants.
4. We excluded participants first testing positive before 11 November 2020 (12 weeks before the Long Covid survey question was implemented) so that we could fully observe participants' self-reported Long Covid experience. Before this date, participants’ Long Covid status 12 weeks post-infection cannot be obtained.
5. To ensure that a first positive swab for SARS-CoV-2 during the study period was likely to represent a first infection, we excluded participants with a first positive swab at CIS enrolment, as the timing of infection could not be determined for these participants.
6. We excluded participants with a positive spike-antibody blood test (excluding any tests after COVID-19 vaccination) or who reported thinking they had had COVID-19 ≥14 days before their first positive swab, as the first observed positive test may have represented a reinfection for these participants
7. Participants aged <16 or >64 years are not considered to be of working-age and were therefore outside the scope of this analysis.
8. Students were outside the scope of this analysis.
9. When analysing long-term absence from work, we excluded study assessments when participants were not in employment and those before 1 October 2021, when the UK Coronavirus Job Retention Scheme (also known as ‘furlough’) was in operation.

**Supplementary Figure 2a.** Unadjusted and adjusted odds ratios for inactivity (excluding retirement) compared with the pre-infection period


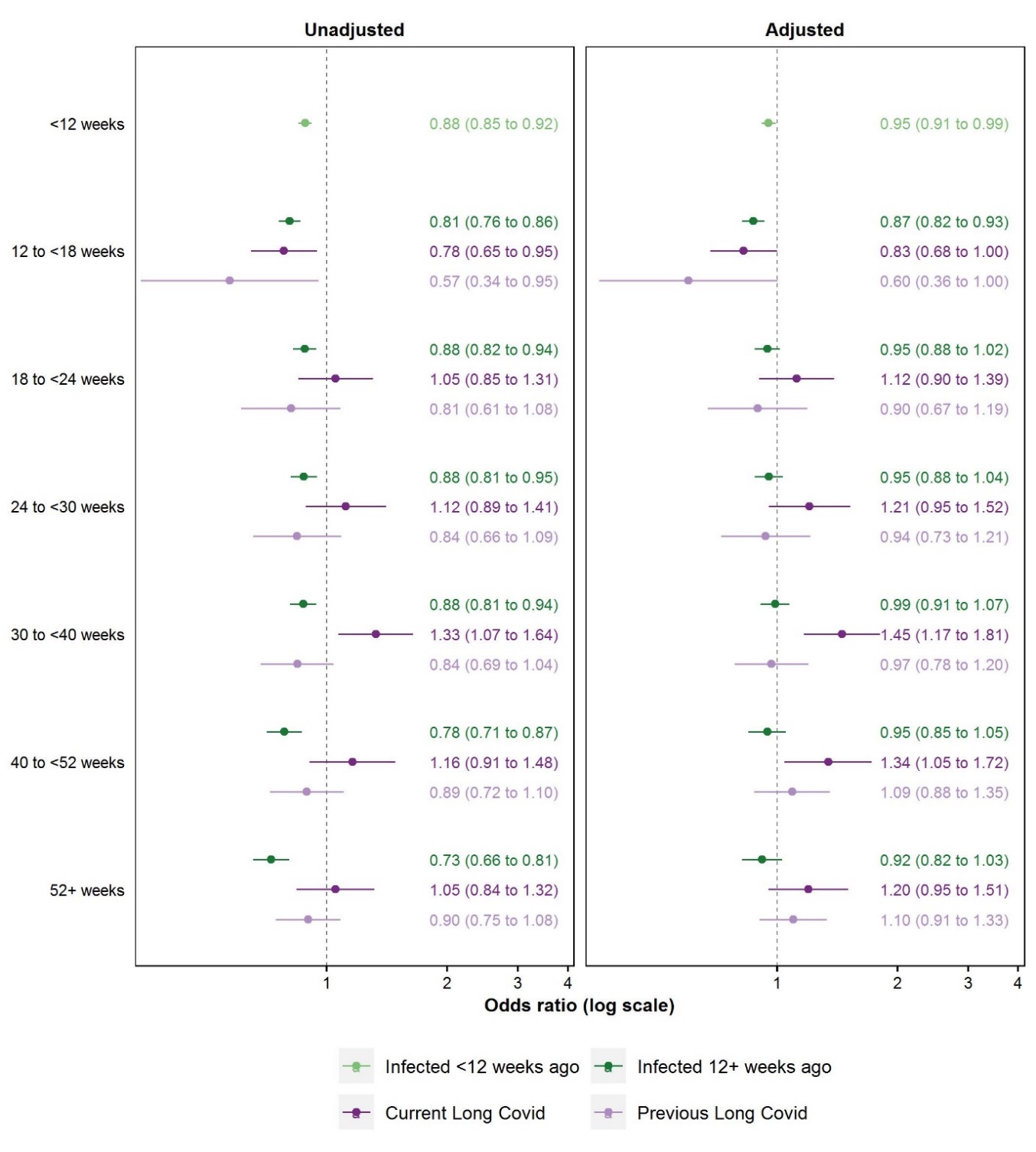


Notes: Both sets of estimates are from conditional logit models. Adjusted estimates are adjusted for calendar day of study assessment, current age, and interactions between calendar day and each of current age, sex, and self-reported health/disability status at survey enrolment.

The adjustment for calendar time appears to have considerable value in reducing bias in the estimates. The unadjusted ORs for inactivity for participants previously infected with SARS-CoV-2 without reporting Long Covid are significantly lower than 1 in all time-since-infection strata, but it is infeasible that being infected with SARS-CoV-2 would increase the likelihood of labour market participation. This is suggestive of bias in the unadjusted estimates, caused by temporal confounding between the risk of infection and background labour market conditions (the likelihood of ever being infected with SARS-CoV-2 increased as the pandemic unfolded, coinciding with increasing employment in the UK labour market from mid-2021).

**Supplementary Figure 2b.** Unadjusted and adjusted odds ratios for long-term (≥4 weeks) absence compared with the pre-infection period


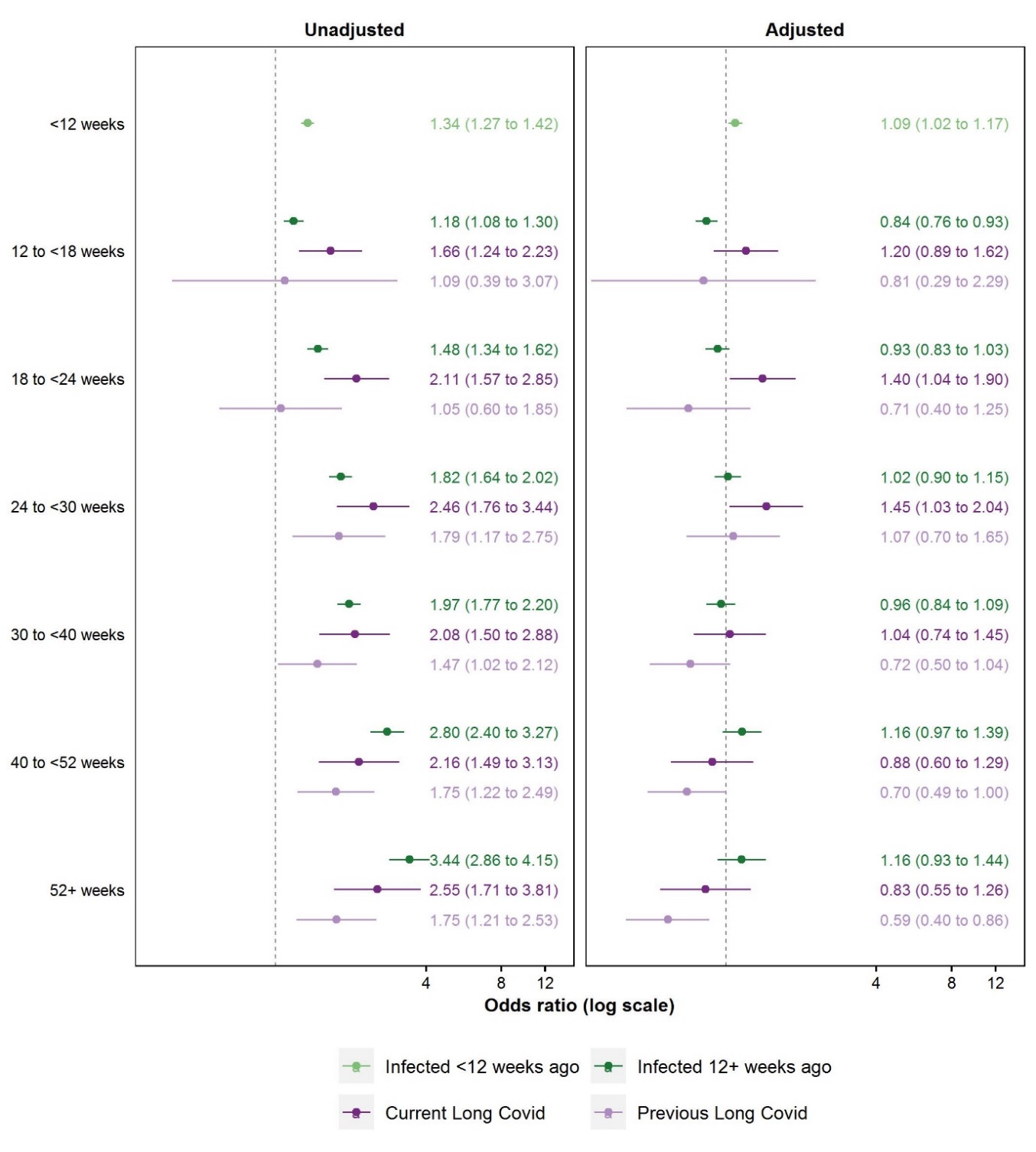


Notes: Both sets of estimates are from conditional logit models. Adjusted estimates are adjusted for calendar day of study assessment, current age, and interactions between calendar day and each of current age, sex, and self-reported health/disability status at survey enrolment. Models were fitted to study assessments from 1 October 2021 when participants were in employment.

**SUPPLEMENTARY APPENDIX 1**

**Survey question used to derive labour market study variables**

All Coronavirus Infection Survey (CIS) participants were asked the following question at enrolment and at each follow-up assessment:

*What is your current work, education or other status, that is, where you spend most of your time? (Select one)*

1. *Employed and currently working (including if on leave or sick leave for less than 4 weeks)*
2. *Employed and currently not working (e.g. on leave due to the COVID-19 pandemic (furloughed); sick leave for 4 weeks or longer; or maternity/paternity leave)*
3. *Self-employed and currently working (include if on leave or sick leave for less than 4 weeks)*
4. *Self-employed and currently not working (e.g. on leave due to the COVID-19 pandemic; sick leave for 4 weeks or longer; or maternity/paternity leave)*
5. *Looking for paid work and able to start*
6. *Not in paid work and not looking for paid work (include doing voluntary work here)*
7. *Retired (include doing voluntary work here)*
8. *Child under 4-5 years not attending nursery, pre-school or childminder*
9. *Child under 4-5 years attending nursery, pre-school or childminder*
10. *4-5 years and older at school/home-school (including if temporarily absent)*
11. *Attending college or other further education provider, including apprenticeships (including if temporarily absent)*
12. *Attending university (including if temporarily absent)*

For this analysis, participants were classified to labour market statuses as follows:

- In employment: response options 1-4
- Long-term absent while in employment: response options 2 and 4
- Unemployed: response option 5
- Labour market inactive (excluding retired): response option 6
- Retired: response option 7
- Student: response options 10-12

**SUPPLEMENTARY APPENDIX 2**

**Sensitivity analyses for labour market inactivity**

We performed several sensitivity analyses for the primary outcome, labour market inactivity:

- First, we restricted the analysis to participants who tested positive for SARS-CoV-2 during follow-up to mitigate against selection effects
- Second, we excluded study assessments when study participants were retired, and therefore ineligible to be otherwise inactive
- Third, we investigated alternative specifications of the restricted cubic spline for modelling calendar time and age

These analyses are illustrated in the following figures. All estimates and inferences are similar to those presented in the main analysis.

Sensitivity analysis 1: Adjusted odds ratios for inactivity (excluding retirement) compared with the pre-infection period, after restricting the analysis population to ever-infected participants


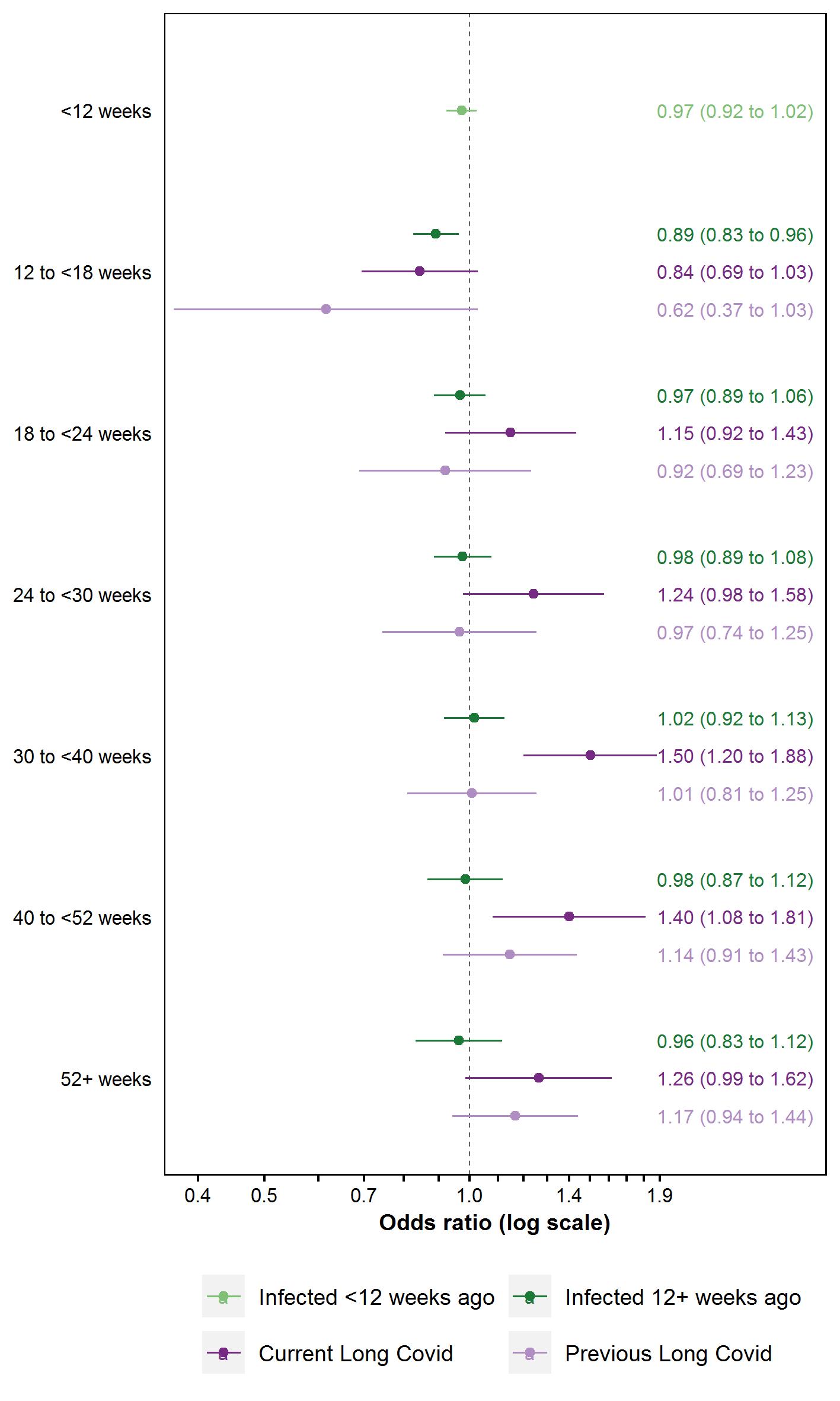


Notes: Estimates are from a conditional logit model adjusted for calendar day of study assessment, current age, and interactions between calendar day and each of current age, sex, and self-reported health/disability status at survey enrolment.

Sensitivity analysis 2: Adjusted odds ratios for inactivity (excluding retirement) compared with the pre-infection period, after excluding study assessments when participants were retired


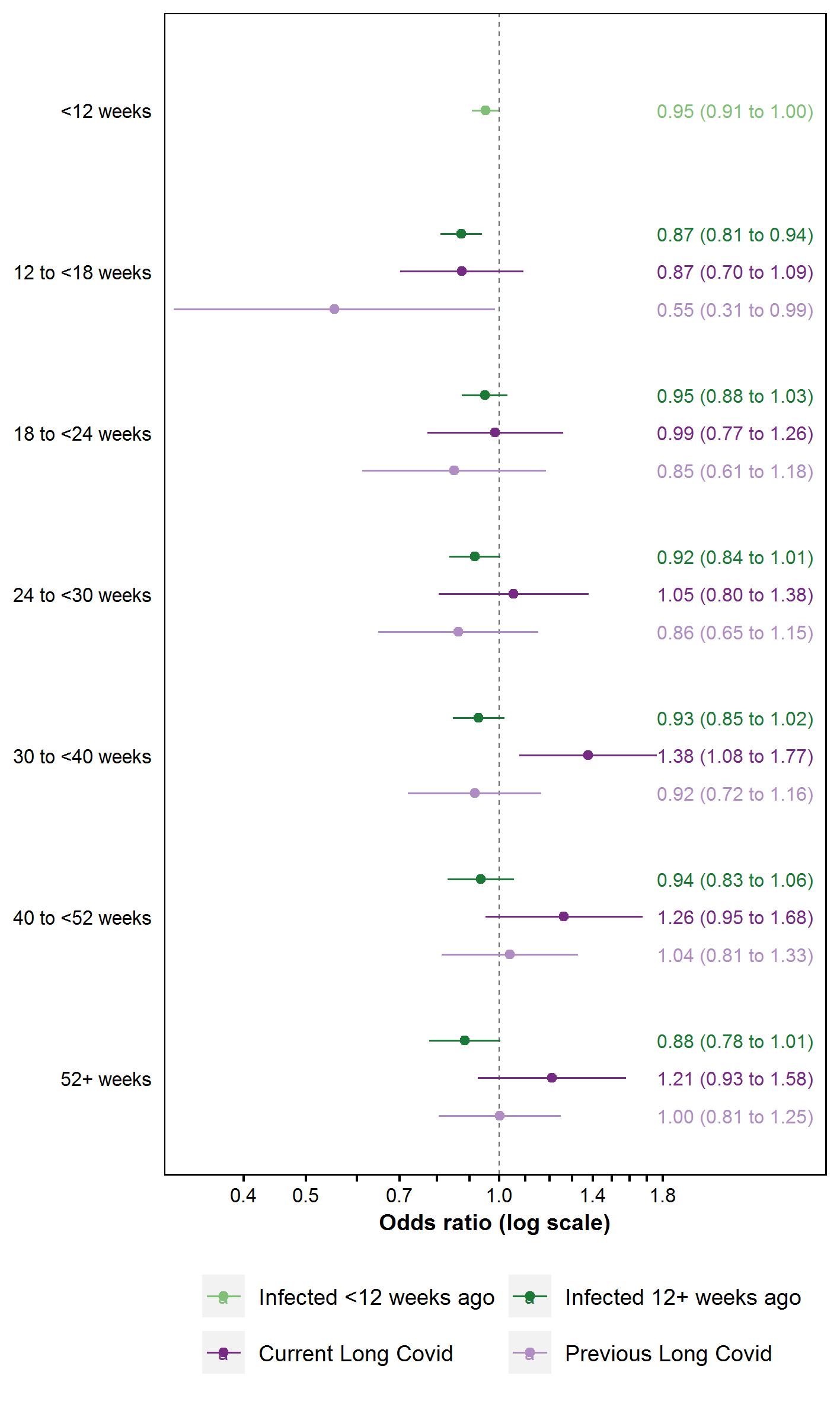


Notes: Estimates are from a conditional logit model adjusted for calendar day of study assessment, current age, and interactions between calendar day and each of current age, sex, and self-reported health/disability status at survey enrolment.

Sensitivity analysis 3a: Adjusted odds ratios for inactivity (excluding retirement) compared with the pre-infection period, after increasing the number of internal knots in splines for calendar time and age from one to two


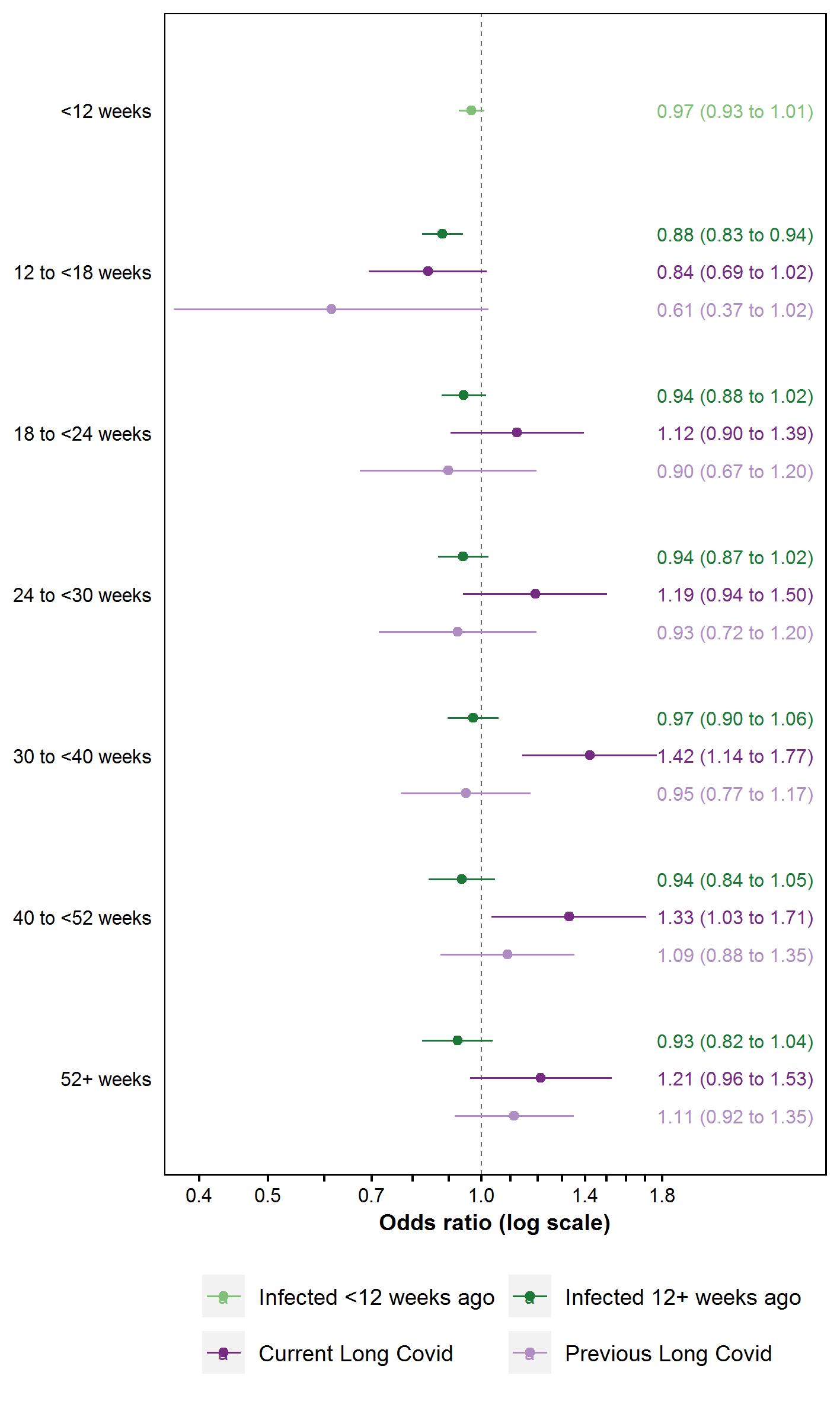


Notes: Estimates are from a conditional logit model adjusted for calendar day of study assessment, current age, and interactions between calendar day and each of current age, sex, and self-reported health/disability status at survey enrolment.

Sensitivity analysis 3b: Adjusted odds ratios for inactivity (excluding retirement) compared with the pre-infection period, after increasing the number of internal knots in splines for calendar time and age from one to three


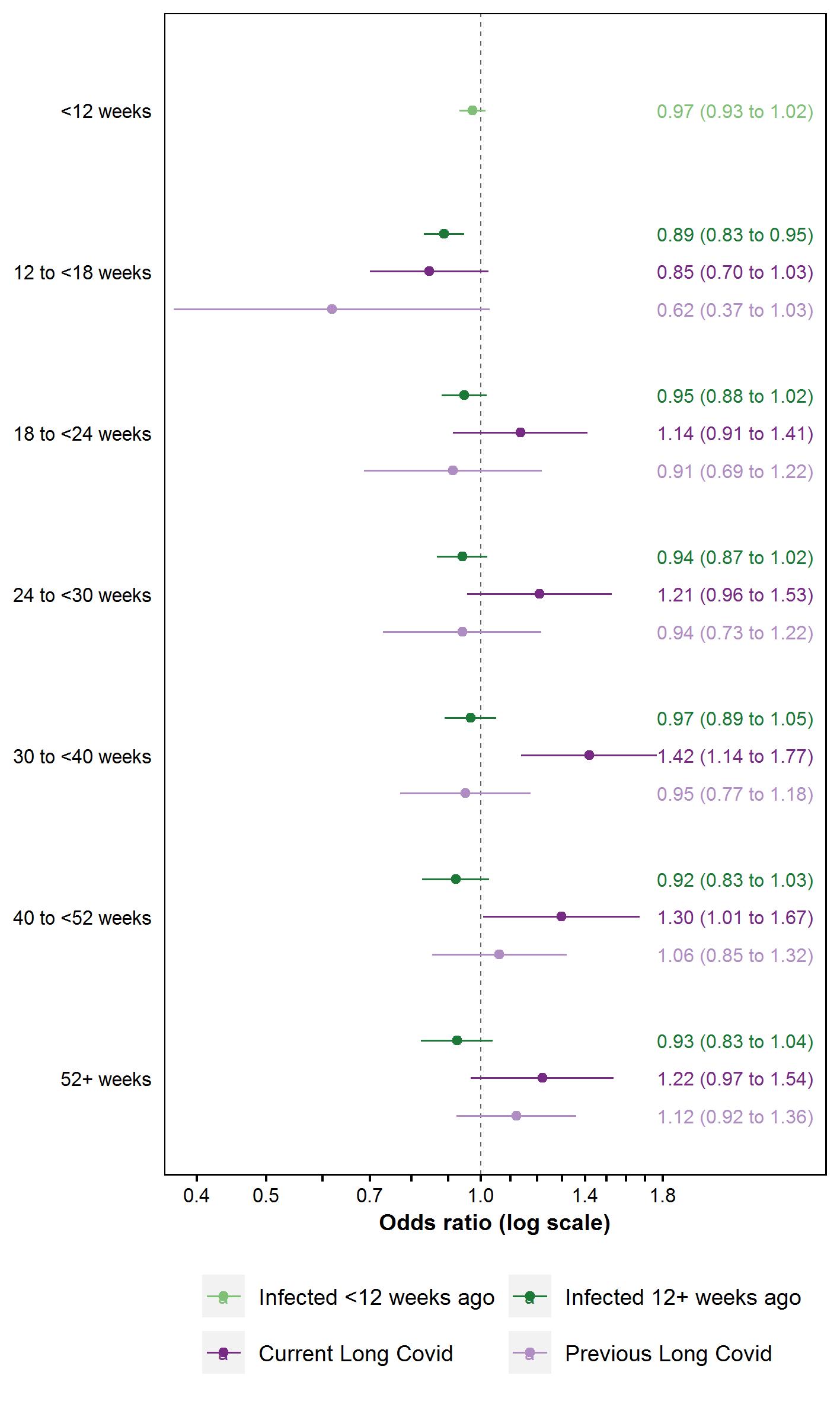


Notes: Estimates are from a conditional logit model adjusted for calendar day of study assessment, current age, and interactions between calendar day and each of current age, sex, and self-reported health/disability status at survey enrolment.

**SUPPLEMENTARY APPENDIX 3**

**Methodology and summary of findings for the heterogeneity analysis**

***Methodology***

We tested for effect modification by socio-demographics at COVID-19 Infection Survey (CIS) enrolment; SARS-CoV-2 reinfection status at each assessment (based on time since first positive swab and number of successive negative tests^1^), which may be associated with Long Covid severity; and mode of data collection to allow for differential exposure misclassification (CIS participants were 30% more likely to report Long Covid if responding remotely rather than face-to-face^2^, perhaps due to stigma associated with the condition^3^). It was possible to test for heterogeneity by labour market attributes (employment sector, SOC Major Group, self-employment status) for long-term absence but not for economic inactivity, as these attributes are only definable for people in employment. For each outcome and each modifier, statistically significant interactions were identified at the 5% level after performing Benjamini-Yekutieli^4^ corrections to p-values to account for multiple comparisons across time-since-infection intervals and levels of modifiers.

***Labour market inactivity***

There was no evidence of heterogeneity in the relationship between Long Covid and inactivity by socio-demographic characteristics, SARS-CoV-2 reinfection status, or data collection mode in any time-since-infection interval **(Supplementary Table 3)**. Despite not reaching the 5% threshold for statistical significance, aORs were consistently numerically highest for participants reporting Long Covid aged 50-64 years.

***Long-term absence***

There was no evidence of heterogeneity in the relationship between Long Covid and long-term absence for any effect modifier in any time-since-infection interval **(Supplementary Table 4)**, except for the presence of underlying health conditions 18 to <24 weeks post-infection (p=0.02). Participants without health conditions who reported Long Covid in this interval were more likely to be long-term absent compared with pre-infection (aOR: 1.96; 95% CI: 1.38 to 2.78), while there was no evidence of difference between the pre- and post-infection periods for participants with health conditions (aOR: 0.58; 95% CI: 0.31 to 1.10).

***References***

^1^ Office for National Statistics. Coronavirus (COVID-19) Infection Survey, characteristics of people testing positive for COVID-19, UK: 19 October 2022. 2022. https://www.ons.gov.uk/peoplepopulationandcommunity/healthandsocialcare/conditionsanddiseases/bulletins/coronaviruscovid19infectionsurveycharacteristicsofpeopletestingpositiveforcovid19uk/19october2022

^2^ Office for National Statistics. Prevalence of ongoing symptoms following coronavirus (COVID-19) infection in the UK: 1 September 2022. 2022. https://www.ons.gov.uk/peoplepopulationandcommunity/healthandsocialcare/conditionsanddiseases/bulletins/prevalenceofongoingsymptomsfollowingcoronaviruscovid19infectionintheuk/1september2022

^3^ Pantelic M, Ziauddeen N, Boyes M, O'Hara ME, Hastie C, Alwan NAA. Long Covid stigma: Estimating burden and validating scale in a UK-based sample. *PLoS One*. 2022;17(11):e0277317. doi: 10.1371/journal.pone.0277317

^4^ Benjamini Y, Yekutieli D. The control of the false discovery rate in multiple testing under dependency. *Ann Stat*. 2001;29(4):1165–1188. doi: 10.1214/aos/1013699998

**SUPPLEMENTARY APPENDIX 4**

**Methodology for estimating labour market inactivity attributable to Long Covid**

***Point estimates***

1. For each time-since-infection stratum ($i$), use the published estimates of the total number of people reporting Long Covid ($n_{i}$) and those who are inactive ($y_{i}$)^1^ to calculate the probability of inactivity among people reporting Long Covid ($p_{i}$):

$$p_{i}={y_{i}}/{n_{i}}$$

| **Column:** | **A** | **B** | **C = B / A** |
| --- | --- | --- | --- |
| **Time since first infection** | **Total with self-reported long COVID (thousands)** | **Number inactive (thousands)** | **Probability of inactivity** |
| 12-17 weeks ago | 123 | 16 | 0.1301 |
| 18-23 weeks ago | 101 | 19 | 0.1881 |
| 24-29 weeks ago | 108 | 20 | 0.1852 |
| 30-39 weeks ago | 151 | 17 | 0.1126 |
| 40-51 weeks ago | 123 | 17 | 0.1382 |
| ≥52 weeks ago | 746 | 112 | 0.1501 |

1. For each time-since-infection stratum, calculate the odds of inactivity ($o_{i}$) among people reporting Long Covid from the probability:

$$o_{i}={p_{i}}/{(1-p_{i})}$$

| **Column:** | **C** | **D = C / (1 - C)** |
| --- | --- | --- |
| **Time since first infection** | **Probability of inactivity** | **Odds of inactivity** |
| 12-17 weeks ago | 0.1301 | 0.1495 |
| 18-23 weeks ago | 0.1881 | 0.2317 |
| 24-29 weeks ago | 0.1852 | 0.2273 |
| 30-39 weeks ago | 0.1126 | 0.1269 |
| 40-51 weeks ago | 0.1382 | 0.1604 |
| ≥52 weeks ago | 0.1501 | 0.1767 |

1. For each time-since-infection stratum, divide the odds of inactivity by the estimated adjusted odds ratio (aOR) for people currently reporting Long Covid ($r_{i}$) in the corresponding time-since-infection stratum; this gives an estimate of the counterfactual odds of inactivity ($\tilde{o}_{i}$) (that is, the odds had those reporting Long Covid not been infected with SARS-CoV-2) assuming the statistical model is correct:

$$\tilde{o}_{i}={o_{i}}/{r_{i}}$$

| **Column:** | **D** | **E** | **F = D / E** |
| --- | --- | --- | --- |
| **Time since first infection** | **Odds of inactivity** | **Adjusted odds ratio for inactivity among people with Long Covid** | **Counterfactual odds of inactivity** |
| 12-17 weeks ago | 0.1495 | 0.83 | 0.1802 |
| 18-23 weeks ago | 0.2317 | 1.12 | 0.2069 |
| 24-29 weeks ago | 0.2273 | 1.21 | 0.1878 |
| 30-39 weeks ago | 0.1269 | 1.45 | 0.0875 |
| 40-51 weeks ago | 0.1604 | 1.34 | 0.1197 |
| ≥52 weeks ago | 0.1767 | 1.20 | 0.1472 |

1. For each time-since-infection stratum, convert the counterfactual odds of inactivity to a counterfactual probability ($\tilde{p}_{i}$):

$$\tilde{p}_{i}={\tilde{o}_{i}}/{(1+\tilde{o}_{i})}$$

| **Column:** | **F** | **G = F / (1 + F)** |
| --- | --- | --- |
| **Time since first infection** | **Counterfactual odds of inactivity** | **Counterfactual probability of inactivity** |
| 12-17 weeks ago | 0.1802 | 0.1527 |
| 18-23 weeks ago | 0.2069 | 0.1714 |
| 24-29 weeks ago | 0.1878 | 0.1581 |
| 30-39 weeks ago | 0.0875 | 0.0805 |
| 40-51 weeks ago | 0.1197 | 0.1069 |
| ≥52 weeks ago | 0.1472 | 0.1283 |

1. For each time-since-infection stratum, multiply the total number of people reporting Long Covid by the counterfactual probability of inactivity; this gives an estimate of the number of people reporting Long Covid who would have been inactive had they not been infected with SARS-CoV-2 ($\tilde{y}_{i}$) assuming the statistical model is correct:

$$\tilde{y}_{i}=n_{i}\tilde{p}_{i}$$

| **Column:** | **A** | **G** | **H = A * G** |
| --- | --- | --- | --- |
| **Time since first infection** | **Total with self-reported long COVID (thousands)** | **Counterfactual probability of inactivity** | **Number inactive if not infected (thousands)** |
| 12-17 weeks ago | 123 | 0.1527 | 19 |
| 18-23 weeks ago | 101 | 0.1714 | 17 |
| 24-29 weeks ago | 108 | 0.1581 | 17 |
| 30-39 weeks ago | 151 | 0.0805 | 12 |
| 40-51 weeks ago | 123 | 0.1069 | 13 |
| ≥52 weeks ago | 746 | 0.1283 | 96 |

1. For each time-since-infection stratum, calculate the difference between the number of people reporting Long Covid who were inactive and the estimated number who would have been inactive had they not been infected with SARS-CoV-2; this gives an estimate of the inactivity attributable to Long Covid ($\Delta_{i}$) assuming the statistical model is correct:

$$\Delta_{i}=y_{i}-\tilde{y}_{i}$$

| **Column:** | **B** | **H** | **I = B - H** |
| --- | --- | --- | --- |
| **Time since first infection** | **Number inactive (thousands)** | **Number inactive if not infected (thousands)** | **Inactivity attributable to Long Covid (thousands)** |
| 12-17 weeks ago | 16 | 19 | -3 |
| 18-23 weeks ago | 19 | 17 | 2 |
| 24-29 weeks ago | 20 | 17 | 3 |
| 30-39 weeks ago | 17 | 12 | 5 |
| 40-51 weeks ago | 17 | 13 | 4 |
| ≥52 weeks ago | 112 | 96 | 16 |

1. Sum the estimated attributable inactivity totals across time-since-infection strata:

$$\Delta=\sum_{i} \Delta_{i}$$

| **Column:** | **I** |
| --- | --- |
| **Time since first infection** | **Inactivity attributable to Long Covid (thousands)** |
| 12-17 weeks ago | -3 |
| 18-23 weeks ago | 2 |
| 24-29 weeks ago | 3 |
| 30-39 weeks ago | 5 |
| 40-51 weeks ago | 4 |
| ≥52 weeks ago | 16 |
|  | **Total: 27** |

***Confidence intervals***

There is uncertainty inherent in both inputs to our estimates: the number of people in the population reporting Long Covid by inactivity status; and the adjusted odds ratios for inactivity by time since first SARS-CoV-2 infection and current Long Covid status. We therefore constructed confidence intervals around our estimates using simulation:

1. For each time-since-infection stratum, take a random draw from the normal distribution with mean equal to the total number of people reporting Long Covid who are inactive, and standard deviation equal to the corresponding standard error.
2. For each time-since-infection stratum, take a random draw from the normal distribution with mean equal to the estimated coefficient for the ‘currently reporting Long Covid’ group from the conditional logit model, and standard deviation equal to the corresponding standard error.
3. For each time-since-infection stratum, take the antilog of the value randomly drawn in step 2 above to obtain the corresponding aOR.
4. Go through steps 1-7 in the ‘point estimates’ subsection, but replacing $y_{i}$ and $r_{i}$ by their randomly drawn values, $y_{i}^{*}$ and $r_{i}^{*}$, to obtain $\Delta^{*}$.
5. Repeat steps 1-4 above a further 9,999 times (i.e., 10,000 iterations in total).
6. Calculate the standard deviations of the sampling distributions of $y^{*}$and $\Delta^{*}$, $s^{y^{*}}$ and $s^{\Delta^{*}}$ respectively. These provide estimates of the standard errors of $y$ and $\Delta$ respectively.
7. Construct 95% confidence intervals around $y$ and $\Delta$:

$y\pm1.96\times s^{y^{*}}$; $\Delta\pm1.96\times s^{\Delta^{*}}$

***References***

^1^ Office for National Statistics. Number of working-age non-students with self-reported long COVID, and those who are economically inactive (excluding retirement), by time since first COVID-19 infection, UK: July 2022. 2023. https://www.ons.gov.uk/peoplepopulationandcommunity/healthandsocialcare/conditionsanddiseases/adhocs/15568numberofworkingagenonstudentswithselfreportedlongcovidandthosewhoareeconomicallyinactiveexcludingretirementbytimesincefirstcovid19infectionukjuly2022
